# Acute exposure to gold nanoparticles aggravates lipopolysaccharide-induced liver injury by amplifying apoptosis via ROS-mediated macrophage-hepatocyte crosstalk

**DOI:** 10.1186/s12951-021-01203-w

**Published:** 2022-01-20

**Authors:** Yongjun Yang, Shijun Fan, Qian Chen, Yongling Lu, Yuanfeng Zhu, Xiaoli Chen, Lin Xia, Qianying Huang, Jiang Zheng, Xin Liu

**Affiliations:** Medical Research Center, Southwest Hospital, Army Military Medical University, Chongqing, 400038 China

**Keywords:** AuNPs, Lipopolysaccharide, Acute liver injury, Apoptosis, Reactive oxygen species

## Abstract

**Background:**

Gold nanoparticles (AuNPs) are increasingly utilized in industrial and biomedical fields, thereby demanding a more comprehensive knowledge about their safety. Current toxicological studies mainly focus on the unfavorable biological impact governed by the physicochemical properties of AuNPs, yet the consequences of their interplay with other bioactive compounds in biological systems are poorly understood.

**Results:**

In this study, AuNPs with a size of 10 nm, the most favorable size for interaction with host cells, were given alone or in combination with bacterial lipopolysaccharide (LPS) in mice or cultured hepatic cells. The results demonstrated that co exposure to AuNPs and LPS exacerbated fatal acute liver injury (ALI) in mice, although AuNPs are apparently non-toxic when administered alone. AuNPs do not enhance systemic or hepatic inflammation but synergize with LPS to upregulate hepatic apoptosis by augmenting macrophage-hepatocyte crosstalk. Mechanistically, AuNPs and LPS coordinate to upregulate NADPH oxidase 2 (NOX2)-dependent reactive oxygen species (ROS) generation and activate the intrinsic apoptotic pathway in hepatic macrophages. Extracellular ROS generation from macrophages is then augmented, thereby inducing calcium-dependent ROS generation and promoting apoptosis in hepatocytes. Furthermore, AuNPs and LPS upregulate scavenger receptor A expression in macrophages and thus increase AuNP uptake to mediate further apoptosis induction.

**Conclusions:**

This study reveals a profound impact of AuNPs in aggravating the hepatotoxic effect of LPS by amplifying ROS-dependent crosstalk in hepatic macrophages and hepatocytes.

**Graphical Abstract:**

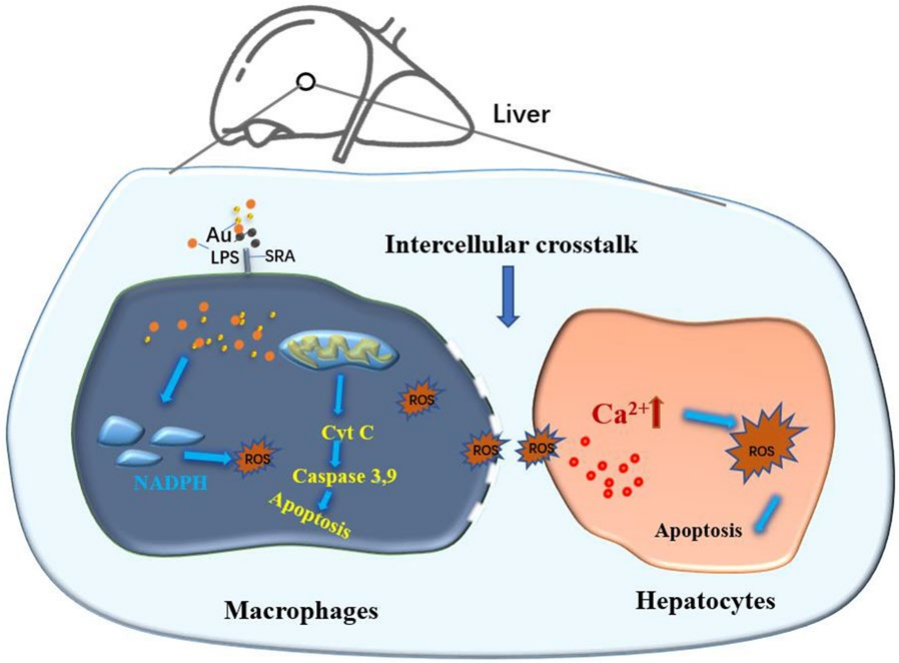

**Supplementary Information:**

The online version contains supplementary material available at 10.1186/s12951-021-01203-w.

## Background

Gold nanoparticles (AuNPs) are among the most extensively investigated metallic nanoparticles in industrial and biomedical fields, owing to their excellent chemical stability, easy functionalization, and inherent photodynamic and photothermal properties [1]. The unique physicochemical properties of AuNPs at the nanoscale, which are expected to augment their reactivity in biological systems, have also raised growing awareness regarding their potential deleterious (e.g. cytotoxic, genotoxic, and immunotoxic) effects [2–4]. It has been extensively shown that the physicochemical features of AuNPs, such as particle size, shape, and surface chemistry, could dramatically alter the mode of cellular contact and uptake of AuNPs, thereby governing their toxic behavior in host cells [5]. However, most of these findings were obtained from studies that examined the individual effects of AuNPs without considering the influence by environmental chemical or biological agents. In fact, AuNPs may contact various chemical substances in the manufacturing process or interact with other biomolecules after administration into the host, resulting in their readily altered toxic behavior. For instance, it was found that AuNPs heteroagglomerated with *E. coli* cells to obtain the capacity to inhibit *C. elegans* growth [6]. Moreover, the mixture of surfactant additives (e.g., Polysorbate 20) with AuNPs produced synergistic toxicity although both were biocompatible when individually examined [7]. These findings indicate the necessity of characterizing the AuNPs-induced toxicity in more complex contexts.

The biomedical use of AuNPs is commonly accompanied by changes in the biological environment of the host. For example, AuNPs have been increasingly explored for diagnosis in bacterial infections [8] or targeted therapy against inflammatory disorders [9], leading to their frequent contact with pathogens, pathogenic molecules, or intrinsic immunomodulatory mediators that are absent in normal physiological conditions. Lipopolysaccharides (LPS) are common pathogenic molecules that make up most of the outer membrane in gram-negative bacteria. A variety of studies have reported the use of AuNPs to detect LPS [10], or AuNPs conjugated with peptides for anti-inflammatory action against LPS [9, 11], thereby increasing the potential interaction or synergistic effect between AuNPs and LPS. Indeed, previous studies have reported that positively charged AuNPs interact with the outer membrane of gram-negative bacteria by binding to LPS [12]. Moreover, the binding of LPS to AuNPs, which was first thought to be a contamination of the AuNPs, augmented its pro-inflammatory properties [13]. These studies implicate the existing interplay between AuNPs and LPS but were primarily performed in vitro, mainly focusing on their application in LPS detection. It remains to be elucidated whether they have synergistic effect which may impose a deleterious impact on the host organs and biological systems.

The liver is the major metabolic organ for microbes, drugs, and other environmental agents in mammals. These substances may cause damage to hepatic cells and induce life-threatening liver injury during metabolism. Oxidative stress is widely considered a key factor for liver injury and is induced by most hepatotoxic agents through direct cellular damage or interaction with inflammation that induces cell death [14]. Emerging evidence suggests that hepatic exposure to LPS induces intercellular networks in major hepatic cells (hepatocytes and hepatic macrophages), thereby intensifying reactive oxygen species (ROS) generation. Excessive ROS release can damage proteins, DNA, and cell membranes by direct oxidation or activating the caspase pathway and inducing apoptosis in hepatic cells [14]. Meanwhile, it is widely thought that AuNPs exert cytotoxicity by inducing oxidative stress [15]. In addition, AuNPs are preferentially accumulated and metabolized in the liver [16]. Thus, they are increasingly used for liver-targeted drug delivery in clinical settings where hepatic infection, inflammation, and oxidative response are common and concomitant [17]. Given the potential interplay between AuNPs and LPS, this study aimed to explore whether acute exposure of AuNPs aggravate the hepatotoxic effect induced by LPS challenge. Therefore, AuNPs with a size of 10 nm, the most favorable size for interaction with host cells [18], were administered alone or in combination with LPS in mice or cultured hepatic cells. Their concurrent exposure to alter hepatic inflammation, oxidative stress, and apoptosis via modulation of macrophage-hepatocyte crosstalk were also investigated.

## Results

### AuNPs aggravate hepatic injuries in LPS-challenged mice

The physicochemical properties of the 10 nm AuNPs were examined before biological studies. As visualized via transmission electron microscope (TEM), the AuNPs were spherical with an average primary size of 10.7 nm (Additional file 1: Fig. S1A). The absorption peak of the AuNPs was observed at 514 nm (Additional file 1: Fig. S1B, Additional file 2: Table S2). Dynamic light scattering (DLS) analysis demonstrated that the hydrodynamic size of AuNPs was 10.2 nm in water, which increased to 21.4 and 25.2 nm in PBS and DMEM solutions, respectively (Additional file 2: Table S2). The ζ-potential of the AuNPs demonstrated a negative surface charge in all solutions (Additional file 2: Table S2). AuNPs at low (1 μg/ml) or high (50 μg /ml) concentrations displayed similar physicochemical properties (Additional file 3: Fig. S2, Additional file 4: Table S3), confirming the reliability of the physicochemical characterization.

**Fig. 1:**
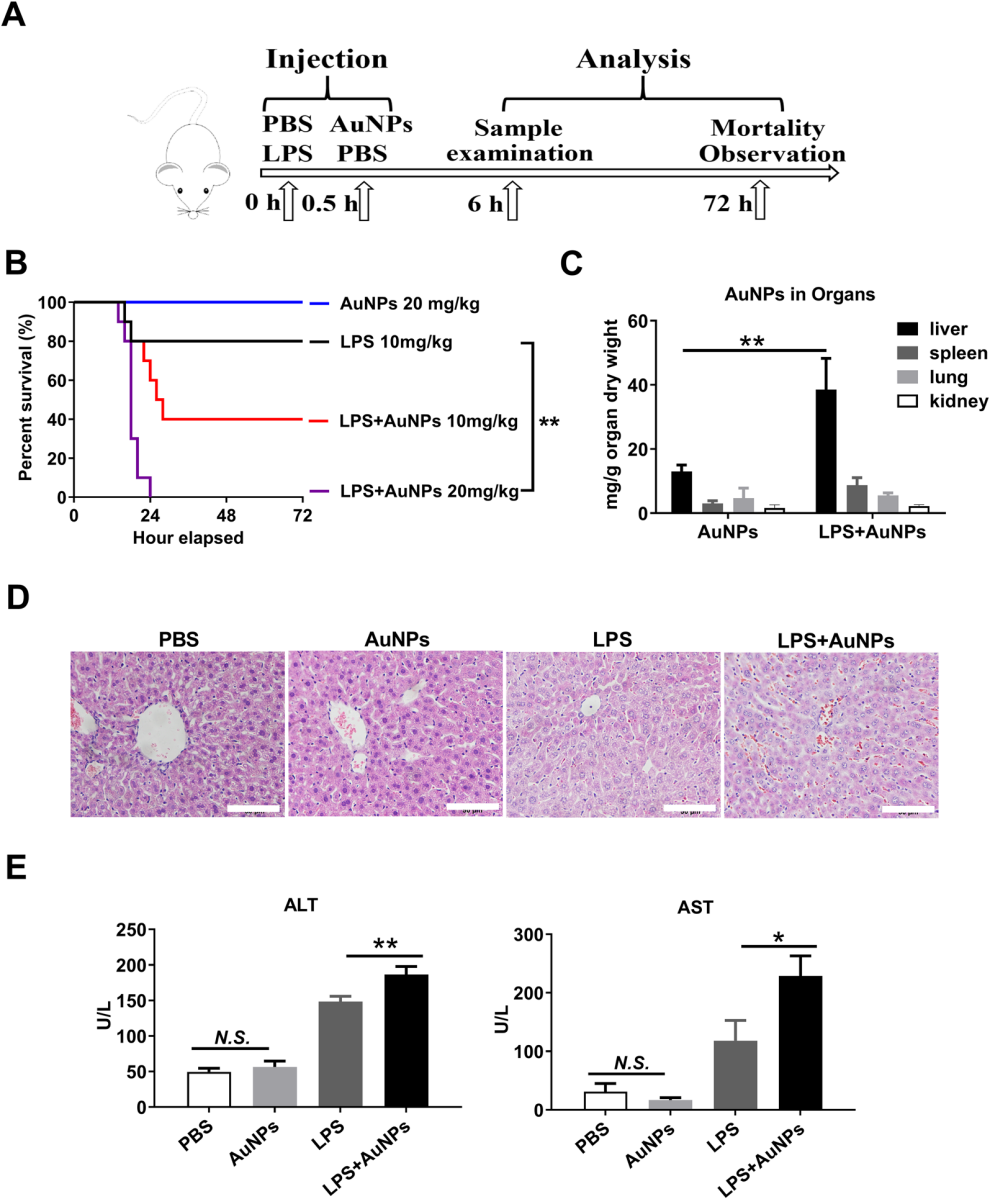
Augmentation in the severity of murine ALI by co-injection with AuNPs and LPS. **A** Study design. **B** The survival curves of mice (n = 10). **C** Quantification of AuNPs in the liver, spleen, lungs, and kidneys of mice via ICP-MS (n = 3). **D** Representative histopathological micrographs of liver sections in mice examined via H&E staining. Magnification, 40× ; scale bar, 50 μm. **E** Serum ALT and AST levels in mice as detected via ELISA. N.S., no significance, * P < 0.05, ** P < *0.01*

Then, AuNPs were administered together with LPS in BALB/c mice to assess their synergistic effect (Fig. 1A). Although bare AuNPs were not lethal, an injection of 10 and 20 mg/kg AuNPs following LPS challenge markedly increased mortality from 20 to 60% and 100%, respectively, suggesting that AuNPs may amplify the toxicity of LPS (Fig. 1B). Next, we examined the organ distribution of the AuNPs. As shown by inductively coupled plasma mass spectrometry (ICP-MS), AuNPs were highly distributed in the liver, and their hepatic distribution was further increased after co-injection with LPS. Only a moderate elevation was observed in the spleen and no substantial alteration in the lungs or kidneys (Fig. 1C). In addition, histopathological examination demonstrated that administration of AuNPs augmented hepatic injury induced by LPS challenge, including severe cell swelling, disorganized structure and elevated infiltration of blood cells in liver tissues in co-administered mice (Fig. 1D). In line with the histopathological observations, serum alanine transaminase (ALT) and aspartate transaminase (AST) levels were significantly upregulated in AuNP- and LPS-co-injected mice than in mice injected with LPS alone (Fig. 1E), which further revealed that AuNPs may augment the hepatotoxic effect of LPS.

### AuNPs synergize with LPS to augment hepatic cell apoptosis without enhancing the pro-inflammatory activity of LPS

**Fig. 2:**
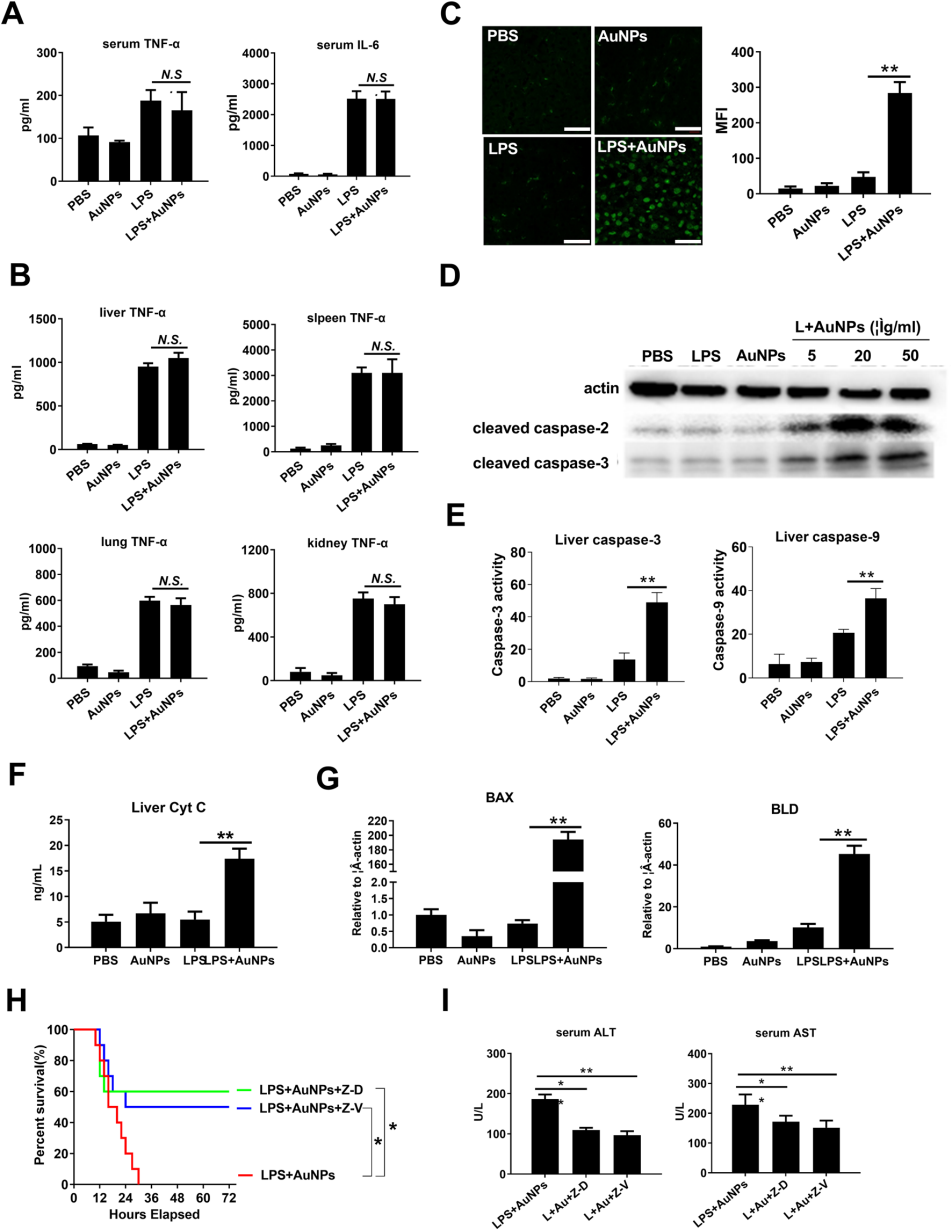
Enhancement of murine hepatic apoptosis instead of inflammation after AuNPs and LPS co-injection. **A**, **B** TNF-α and IL-6 quantification in serum or homogenates of the liver, spleen, lungs, and kidney of mice. **C** Apoptosis detection in liver sections via TUNEL staining. Scale bar, 50 μm. **D** Western blot analysis of cleaved caspase-2 and caspase-3 in liver tissue. **E**, **F** Detection of caspase-3, caspase-9 and Cyt C in liver tissues. **G** Detection of mRNA expression of BAX and BLD in liver tissues via RT-PCR. **H**, **I** Mice were pre-treated with Z-DEVD-FMK (Z-D) or Z-VAD (OMe)-FMK (Z-V) and then challenged with AuNPs and LPS. Their survival (n = 10) was observed and serum ALT and AST levels were detected. Samples were obtained 6 h post-challenge (n = 3). *N.S.,* no significance. * P < 0.05, ** P < *0.01*

Upregulation of inflammation is thought to be a common cause of liver injury [19]. Thus, we examined whether AuNPs enhanced the pro-inflammatory activity of LPS. Nanoparticles are easily contaminated by pyrogens, such as LPS, resulting in a false effect that induces inflammation [20]. Using the limulus amebocyte lysate (LAL) assay, we demonstrated that AuNPs were LPS-free upon suspension in H2O, PBS, and DMEM (Additional file 5: Fig. S3A). Moreover, TNF-α and IL-6 levels were not upregulated upon AuNP treatment in cultured macrophages (Additional file 5: Fig. S3B). In vivo, mice injected with AuNPs alone displayed basal levels of serum LPS (Additional file 5: Fig. S3C) or pro-inflammatory cytokines (Additional file 5: Fig. S3D), which were comparable to those in PBS-injected mice. These results not only exclude potential LPS contamination in AuNPs, but also suggest that AuNPs are unable to independently induce inflammation. Next, we determined whether the pro-inflammatory status in mice was altered after co-injection of AuNPs and LPS. Strikingly, serum TNF-α and IL-6 levels were not increased in the combination-injected mice than in the LPS-injected mice (Fig. 2A). Consistently, TNF-α levels in the homogenates of major organs subject to LPS injection were also not further upregulated by additional AuNP administration. Moreover, as shown by westernblot analysis in liver homogenates, the protein levels of TNF-α and IL-6 as well as the phosphorylated ERK (a proinflammation signaling molecule) and TLR4 (a proinflammatory pattern recognition receptor of LPS) in liver were not increased by AuNPs co-injection (Additional file 6: Fig. S4). Therefore, AuNPs may not amplify the hepatotoxicity of LPS by augmenting systemic or local inflammation.

Apoptosis induction is another critical pathophysiological mechanism underlying liver injury [21]. By performing a TdT-mediated dUTP nick-end labeling (TUNEL) assay in liver tissue sections, we found that AuNPs alone did not induce higher apoptosis in hepatic cells than PBS injection. Conversely, a pronounced increase in apoptosis was detected in the liver tissues of mice after co-injection with AuNPs and LPS, compared to intermediate levels of hepatocyte apoptosis after injection with LPS only (Fig. 2C). AuNPs also activated the pro-apoptotic caspase cascade, as shown by their dose-dependent synergy with LPS to upregulate the protein levels of cleaved caspase-2 and caspase-3 in liver homogenates (Fig. 2D). Meanwhile, the activity of hepatic caspase-3 and caspase-9 was also significantly enhanced by combined AuNPs and LPS treatment (Fig. 2E), compared to no substantial change observed in the kidneys or the lungs (Additional file 7: Fig. S5A, B). Additionally, the protein levels of cytochrome c (Fig. 2F) and the mRNA expression of BCL2 Associated X (BAX) and BH3 interacting domain death agonist (BLD) (Fig. 2G) in the liver were remarkably upregulated in mice co-injected with AuNPs and LPS, which further indicated that AuNPs and LPS activated the intrinsic apoptosis pathway (Fig. 2G). Next, mice were administered the caspase-3 inhibitor Z-DEVD-FMK or the pan-caspase inhibitor Z-VAD (OMe)-FMK before being subjected to AuNPs and LPS challenge. When apoptosis was inhibited in mice, their mortality was significantly decreased (Fig. 2H), and the elevated serum ALT and AST levels were also markedly reduced (Fig. 2I). Altogether, these results indicate that AuNPs may amplify the hepatotoxic effect of LPS by cooperatively promoting intrinsic apoptosis.

### AuNPs and LPS enhance macrophage-hepatocyte crosstalk to mediate hepatic apoptosis

**Fig. 3:**
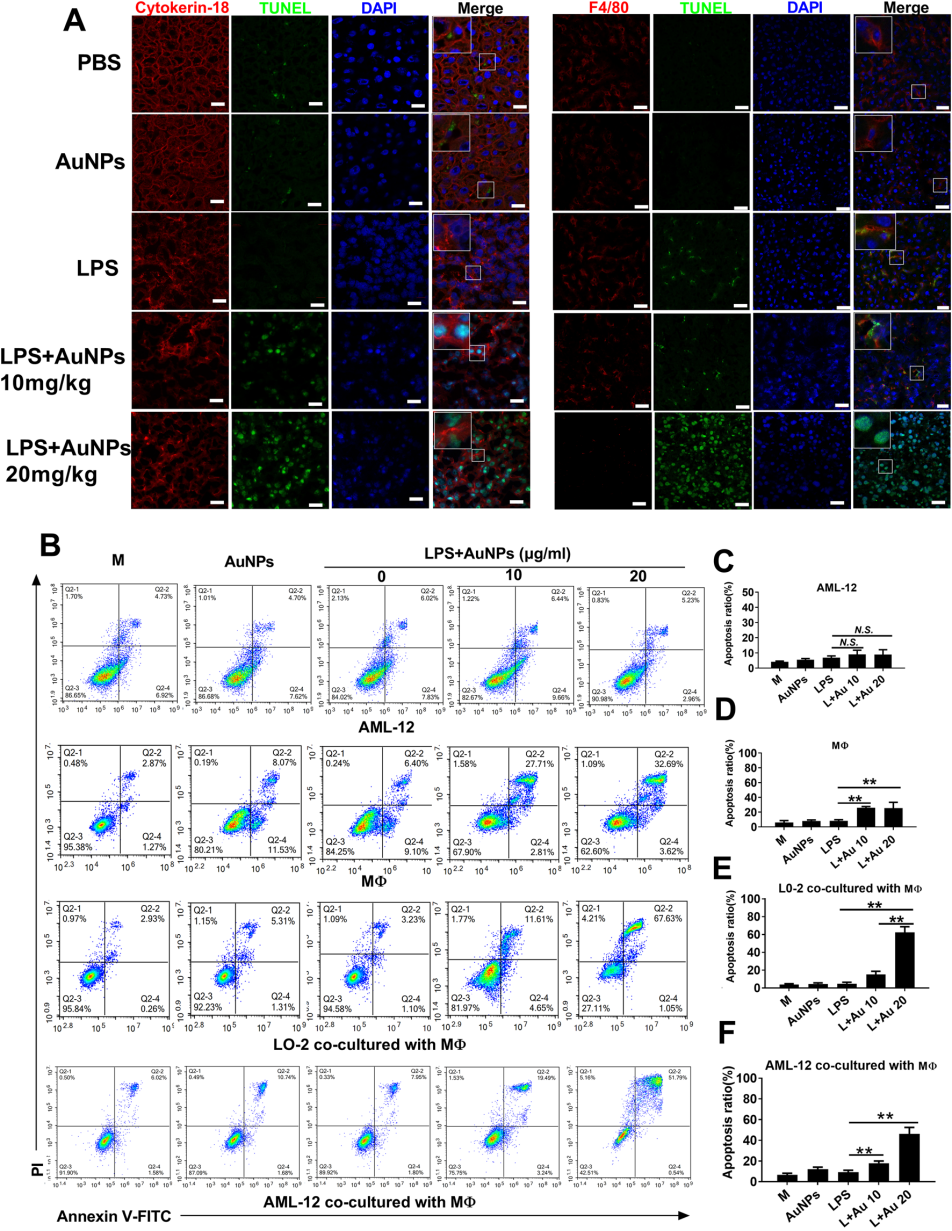
Apoptosis detection in hepatic macrophages and hepatocytes after AuNPs and LPS co-treatment. **A** Distribution of TUNEL-positive signals and immunofluorescent staining of F4/80 or cytokerin-18 in liver cryosections. Scale bar, 50 μm. **B** Flow cytometry analysis of apoptosis via Annexin V-FITC/PI double staining in independently cultured AML-12 cells and murine peritoneal macrophages (MΦ) or a co-culture of L0-2 cells or AML-12 cells with MΦ in a transwell system. **C**–**F** Quantification of the apoptotic ratio in B) (n = 3). N.S., no significance. * P < 0.05, ** P < *0.01*

Liver injury is commonly initiated by the crosstalk between macrophages and hepatocytes [22]. Therefore, we examined whether hepatic apoptosis induced by the administration of AuNPs and LPS was mediated by the interaction between macrophages and hepatocytes. As shown in Fig. 3A, TUNEL staining was also distributed in F4/80 (macrophage marker) and cytokerin-18 (hepatocyte marker) expressing cells in the liver sections of mice, indicative of apoptosis in both hepatocytes and hepatic macrophages. Although the correlation coefficients were low between TUNEL staining and fluorescent probes for cytokerin-18 or F4/80, TUNEL and DAPI staining was highly overlapped (Additional file 8: Fig. S6). Moreover, co-injection of AuNPs and LPS increased the amount of TUNEL staining and induced cell damage, as shown by the decreased fluorescence of F4/80 and cytokerin-18 accompanied by a deformation in the tissue structure of the liver and abnormal morphology in hepatocytes and hepatic macrophages. Next, apoptosis was also detected directly in cultured hepatic cells via PI/annexin staining. Unexpectedly, apoptosis was not upregulated in independently cultured AML-12 cells, despite co-treatment with AuNPs and LPS (Fig. 3B, C). Likewise, apoptosis was not induced in other parenchymal cells, including HMVECs and mouse fibroblasts, following identical treatments (Additional file 9: Fig. S7A). However, apoptosis was enhanced in cultured murine peritoneal macrophages (Fig. 3B, D) and murine peripheral lymphocytes or neutrophils (Additional file 9: Fig. S7B) stimulated by AuNPs and LPS, suggesting that AuNPs and LPS may selectively promote apoptosis in non-parenchymal hepatic cells. To further address whether AuNPs and LPS induced apoptosis in hepatocytes by promoting intercellular crosstalk between macrophages and hepatocytes, murine peritoneal macrophages and AML-12 cells were co-cultured in Transwell chambers. The percentage of apoptotic AML-12 cells or L0-2 cells (a human normal hepatocyte cell line introduced as the same cell type of AML-12) was markedly increased when they were co-cultured with murine peritoneal macrophages and stimulated with AuNPs and LPS (Fig. 3B–F). In contrast, apoptosis was not increased in AML-12 cells co-cultured with HEK293 cells pre-stimulated with AuNPs and LPS (Additional file 10: Fig. S8A) or in HMVECs (a control type of human parenchymal cells for AML-12 and L0-2 cells) co-cultured with AuNPs- and LPS-treated macrophages (Additional file 10: Fig. S8B). Overall, these results suggest that AuNPs and LPS do not only directly promote apoptosis in hepatic macrophages but may also indirectly induce apoptosis in hepatocytes by enhancing the crosstalk between hepatic macrophages and hepatocytes.

### Depletion of macrophages alleviates hepatic injury induced by co-treatment with AuNPs and LPS

**Fig. 4:**
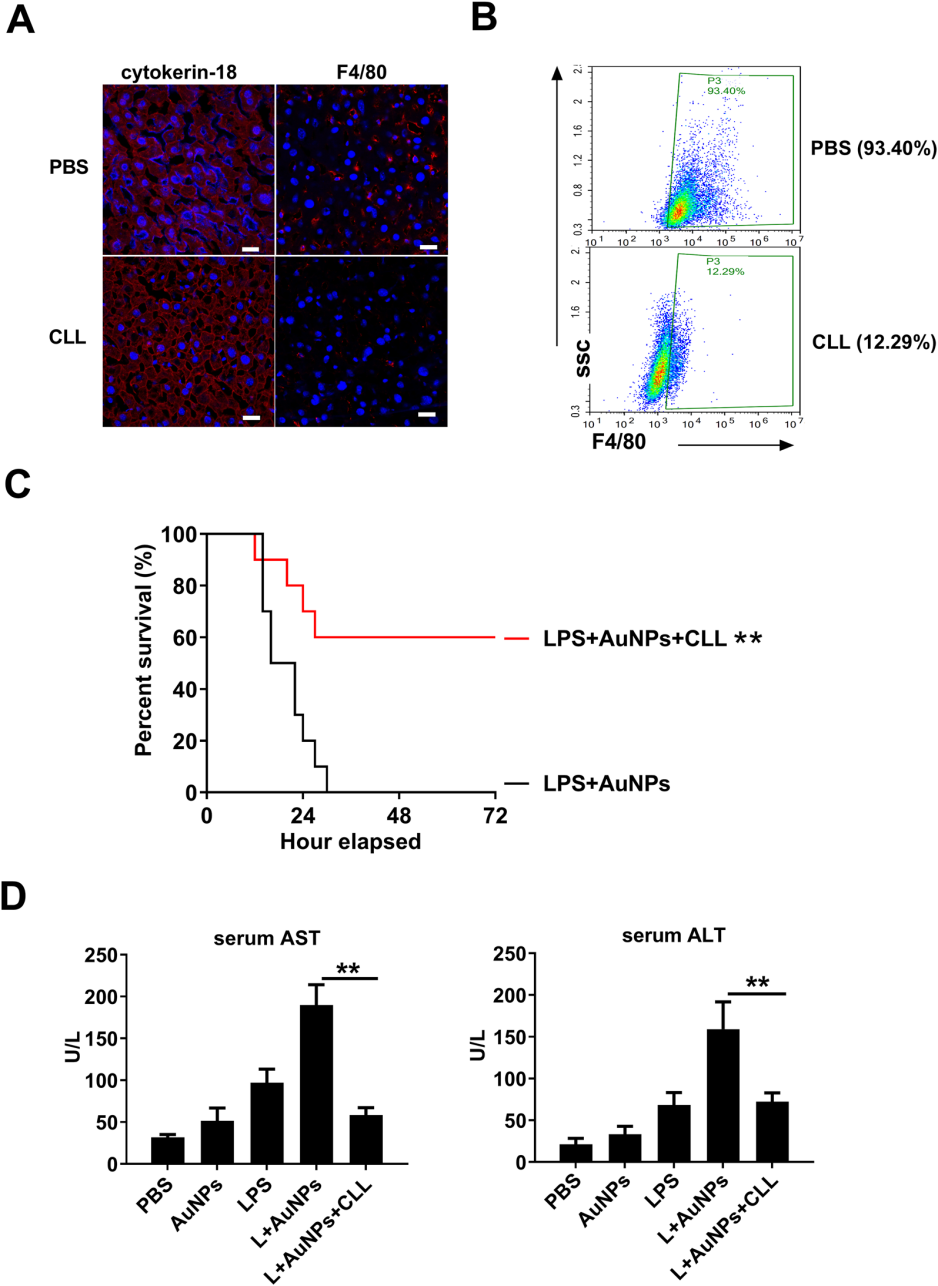
Depletion of macrophages abrogates ALI induced by co-injection with AuNPs and LPS. **A**, **B** Determination of macrophage depletion by detection of F4/80 stained cells in liver sections (**A**) or in peripheral blood cells (**B**) 24 h after injection. **C**, **D** Mice were administrated with PBS or CCL and then subjected to AuNPs and LPS challenge. Their survival (n = 10) was observed for 72 h (**C**), and their serum ALT and AST levels were detected 6 h after challenge (**D**) (n = 3). ** P < *0.01*

To further address the role of hepatic macrophages in mediating hepatic apoptosis and injury after AuNPs and LPS co-challenge, hepatic macrophages were depleted via injection with clodronate liposomes. A marked decrease in F4/80 staining was detected in peripheral blood cells (Fig. 4A) and in the liver tissues (Fig. 4B) of mice after clodronate liposome injection, confirming the widespread loss of macrophages. As expected, mortality was significantly reduced in macrophage-depleted mice (40%) compared to normal mice (100%) when both were injected with AuNPs and LPS (Fig. 4C). Concomitantly, the elevation of serum ALT and AST levels (Fig. 4D) due to AuNPs and LPS co-challenge was also markedly suppressed in macrophage-depleted mice, suggesting that hepatic macrophages, either resident or infiltrated, are responsible for mediating the lethal hepatic injury in mice induced by AuNPs and LPS.

### AuNPs enhance ROS production and trigger apoptosis in LPS-stimulated macrophages

**Fig. 5:**
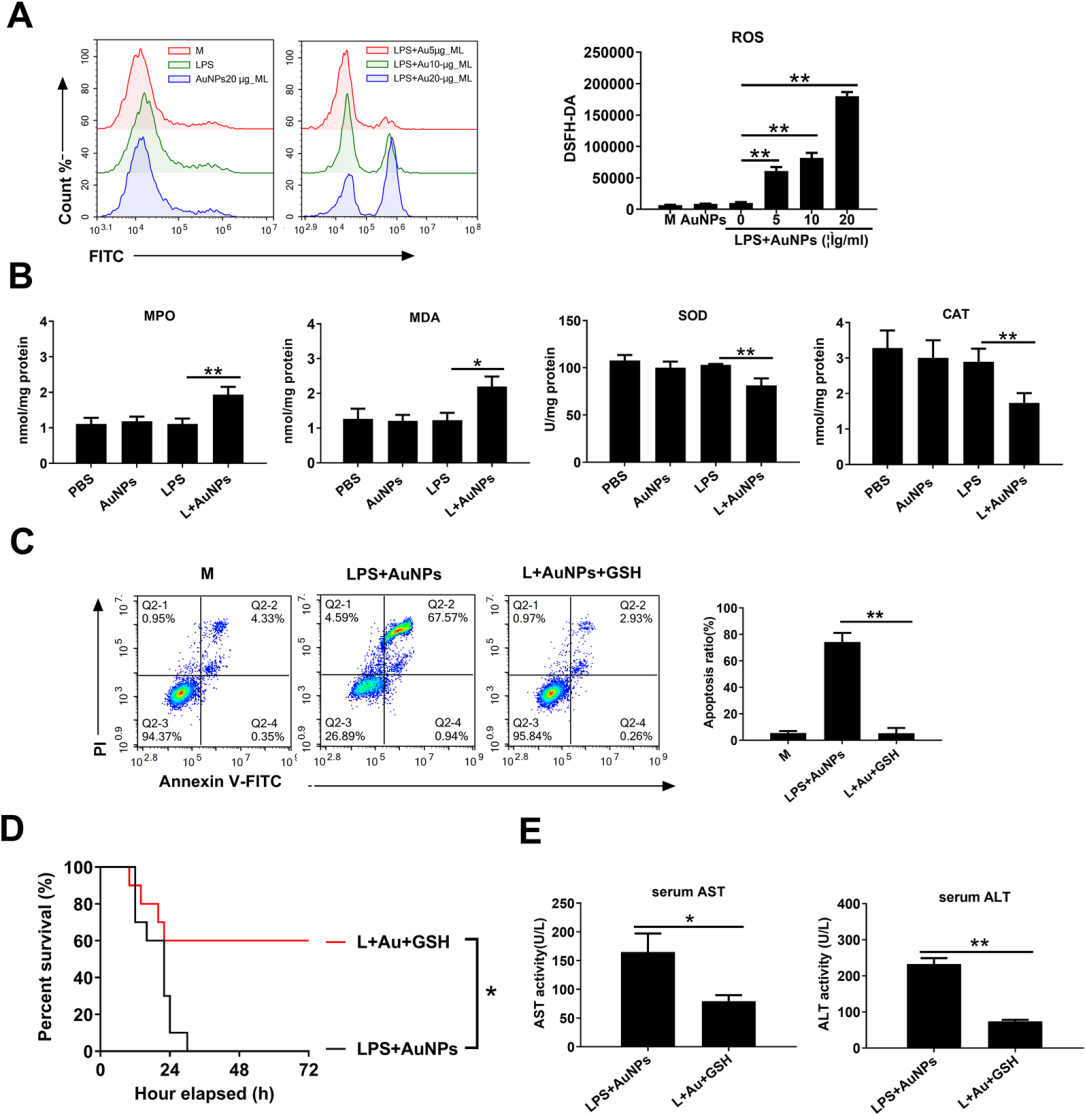
AuNPs enhance ROS production to trigger apoptosis in LPS-stimulated macrophages. **A** Murine peritoneal macrophages were treated with 20 μg/ml AuNPs, 10 ng/ml LPS, or a combination of LPS and AuNPs (5–20 μg/ml) for 4 h. Intracellular ROS levels were detected via DCFH-DA staining. **B** Mice were injected with PBS or 10 mg/kg LPS and subsequently with PBS or 20 mg/kg AuNPs for 6 h. Serum levels of MIDA, MPO, SOD, and CAT were detected. **C** Murine peritoneal macrophages were treated with 20 μg/ml AuNPs and 10 ng/ml LPS with or without GSH, and apoptosis was detected via Annexin V-FITC/PI staining. **D**, **E** Mice were injected with 10 mg/kg LPS and 20 mg/kg AuNPs with or without GSH (**D**). Their survival was observed for 72 h (n = 10) and blood samples were collected 6 h after injection for the detection of serum levels of ALT and AST (**E**) (n = 3). N.S., no significance, *: P < 0.05, **: P < *0.01*

The elevation of intracellular ROS is a hallmark of oxidative stress and is essential for inducing intrinsic apoptosis [23]. Therefore, we examined whether AuNPs and LPS upregulated ROS generation and triggered apoptosis in macrophages. As shown by DCFH-DA staining, the basal ROS level in murine peritoneal macrophages was not altered by AuNP treatment alone. However, AuNPs synergized with LPS to robustly upregulate ROS generation (Fig. 5A). We next evaluated whether co-administration of AuNPs and LPS induced liver injury in mice by favoring the oxidant status instead of the antioxidant status. As expected, serum Malondialdehyde (MIDA) and myeloperoxidase (MPO), two indicators of circulatory oxidative status, were further upregulated after AuNP administration in LPS-challenged mice. In contrast, serum superoxide dismutase (SOD), catalase (CAT), two key intrinsic antioxidant enzymes, were markedly downregulated after co-treatment with AuNPs and LPS (Fig. 5B). Furthermore, the increase in the number of apoptotic macrophages due to AuNP and LPS co-treatment was remarkably abolished by GSH, an intrinsic ROS scavenger (Fig. 5C). Administration of GSH before AuNPs and LPS challenge also resulted in a marked increase in survival (Fig. 5D) and a significant decrease in serum ALT and AST levels (Fig. 5E). These data suggest that co-treatment with AuNPs and LPS enhances ROS production, facilitating macrophage apoptosis and amplifying ALI in mice.

### AuNPs and LPS activated NOX2 in macrophages to mediate ROS generation and apoptosis

**Fig. 6:**
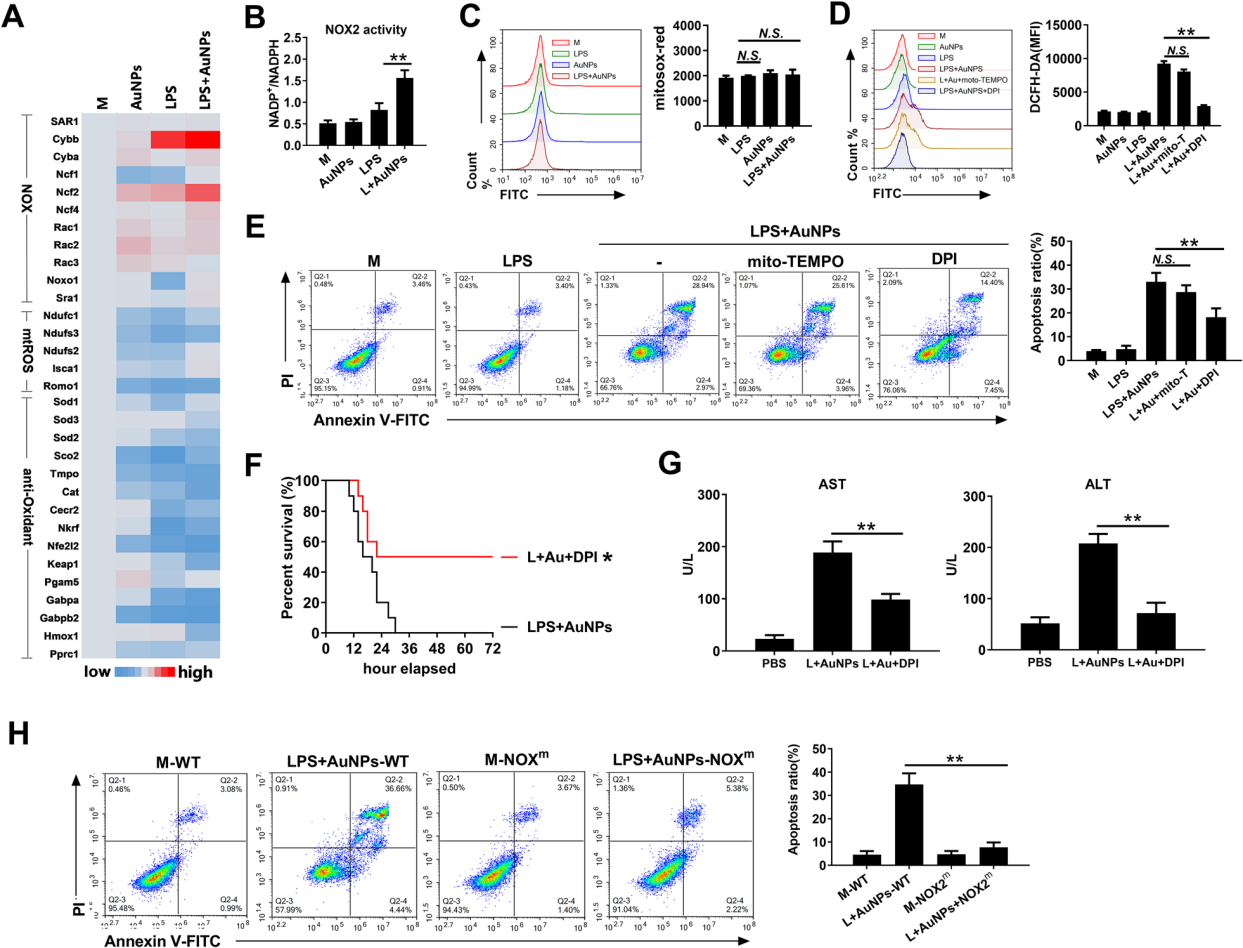
AuNPs and LPS activate NOX2 to induce ROS generation and apoptosis. **A**–**C** Murine peritoneal macrophages were treated with 20 μg/ml AuNPs and 10 ng/ml LPS separately or in combination for 4 h. Gene transcription was analyzed via RNA-seq analysis (**A**). NOX2 activity was analyzed by calculating the NADP + /NADPH ratio (**B**). mtROS level was examined via MitoSOX red staining (**C**). **D**, **E** Murine peritoneal macrophages were treated with LPS and AuNPs with or without mito-TEMPO or DPI for 2 h. Cytosolic ROS levels were detected via DCFH-DA staining (**D**). Apoptosis was analyzed via AnnexinV-FITC/PI staining (**E**). **F**, **G** BALB/c mice were injected with 10 mg/kg LPS and 20 mg/kg AuNPs with or without the administration of GSH. Their survival (n = 10) was observed for 72 h (**F**). Blood samples were collected 6 h after injection for the detection of serum ALT and AST levels (**G**). **H** Peritoneal macrophages from wild-type (WT) or NOX2-mutant (NOXm) C57/BL6 mice were treated with 20 μg/ml AuNPs and 10 ng/ml LPS for 4 h. Apoptosis was analyzed via Annexin V-FITC/PI staining. N.S., no significance, *: P < 0.05, **: P < *0.01*

To further characterize the mechanism by which AuNPs and LPS alter cellular redox status, we performed RNA-seq analysis in macrophages. The results demonstrated that transcripts of the NOX2 complex components, including cytochrome b-245 beta chain (cybb), cytochrome b-245 alpha chain (cyba), and neutrophil cytosolic factor 2 (ncf2), were upregulated after treatment with AuNPs and LPS. Meanwhile, antioxidant genes, including sod, cat, synthesis of cytochrome c oxidase (sco_2_), and GA binding protein transcription factor subunit beta 2 (gabpb2), were significantly downregulated after co-treatment with AuNPs and LPS compared to treatment with LPS alone (Fig. 6A). Consistent with the RNA-seq data, a remarkable increase in NOX2 activation was induced by co-treatment with AuNPs and LPS in macrophages (Fig. 6B). Conversely, mtROS generation did not significantly increase (Fig. 6C). The elevated ROS production induced by AuNPs and LPS was also significantly inhibited by the NOX inhibitor diphenyleneiodonium chloride (DPI), but not by the mtROS inhibitor mito-TEMPO (Fig. 6D). Likewise, apoptosis in macrophages was suppressed by DPI instead of mito-TEMPO (Fig. 6E). In vivo, administration of DPI significantly improved survival (Fig. 6F) and reduced liver injury (Fig. 6G) in mice co-injected with AuNPs and LPS. To further confirm the role of NOX2 in mediating apoptosis in macrophages, the extent of apoptosis was compared in peritoneal macrophages isolated from wild-type or NOX2 mutant C57/BL6 mice (Additional file 11: Fig. S9). In response to AuNPs and LPS co-treatment, the apoptosis ratio was remarkably reduced in macrophages of NOX2-mutant mice compared to that in wild-type mice (Fig. 6H). Therefore, the results demonstrate NOX2-dependent ROS generation by AuNPs and LPS co-treatment in murine macrophages, which mediate apoptosis and hepatic injury.

### AuNPs and LPS increase extracellular ROS production in cultured macrophages to promote ROS generation and apoptosis in hepatocytes

**Fig. 7:**
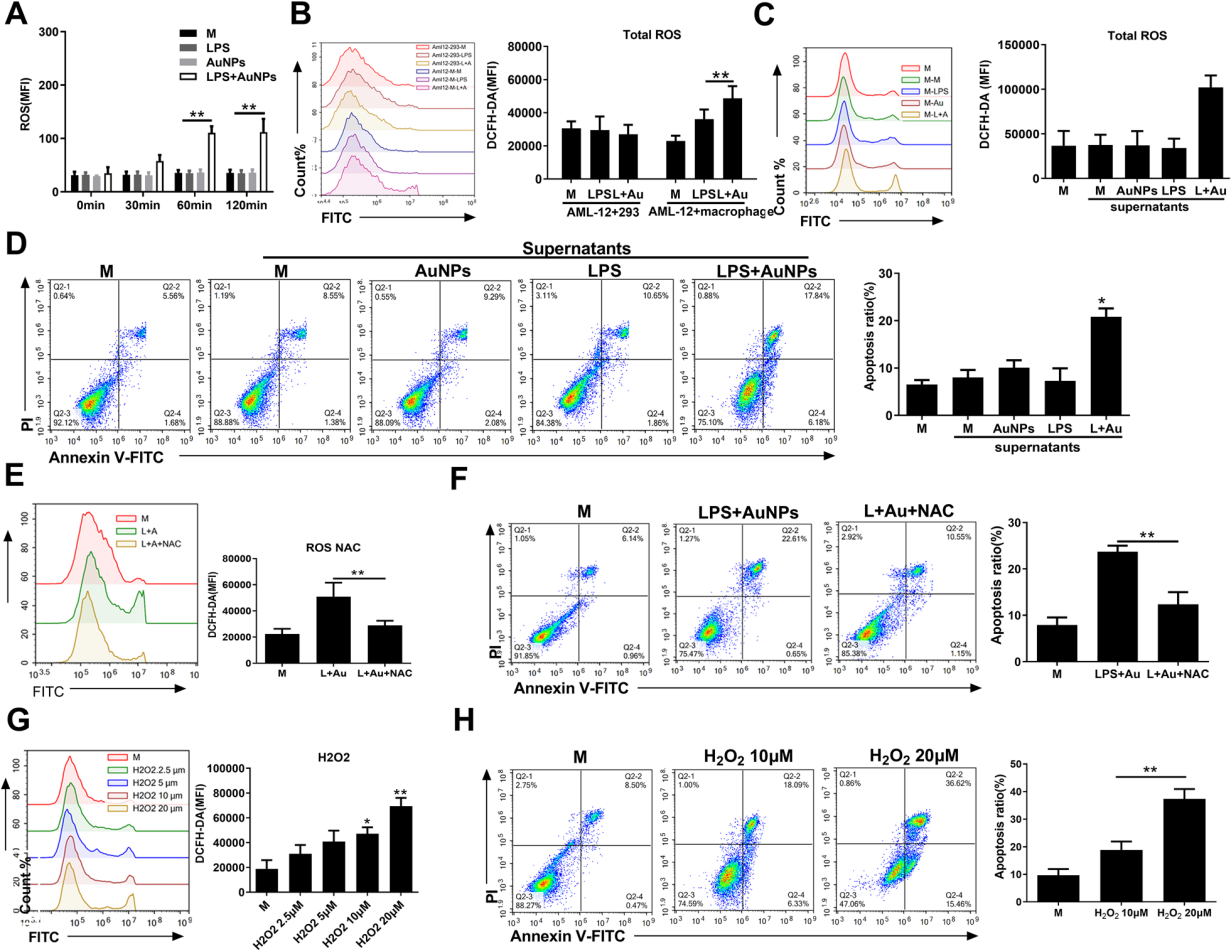
AuNPs and LPS increase extracellular ROS production in cultured macrophages to promote ROS generation and apoptosis in hepatocytes. **A** Detection of ROS in the supernatant of macrophage cultures treated with AuNPs and LPS for 0–120 min. **B** ROS detection in AML-12 cells co-cultured with LPS and AuNPs treated macrophages or HEK293 cells. **C**, **D** Detection of total ROS (**C**) and apoptosis (**D**) in AML-12 cells treated with supernatants from macrophages treated with AuNPs and LPS separately or in combination for. **E**, **F** Detection of total ROS (**E**) and apoptosis (**F**) in AML-12 cells treated by AuNPs and LPS conditioned macrophage supernatants with or without NAC for 4 h. Treatment with NAC inhibits ROS generation. **G**, **H** Detection of total ROS (**G**) and apoptosis (**H**) in AML-12 cells treated by H_2_O_2_. Cell treatment was 4 h unless indicated (n = 3). *: P < 0.05, **: P < *0.01*

We next examined whether AuNPs and LPS upregulated extracellular ROS levels in cultured macrophages, validating their involvement in mediating macrophage-hepatocyte crosstalk and apoptosis induction in hepatocytes. First, the ROS levels in the supernatants of macrophages cultured in vitro were significantly elevated by 60 min or more after AuNP and LPS co-treatment, compared to no substantial increase upon treatment with either agent alone (Fig. 7A). By contrast, ROS were not upregulated in either DMEM containing AuNP and LPS (without macrophages) or the supernatants of individual AML-12 cells exposed to AuNP and LPS (Additional file 12: Fig. S10), suggesting that AuNPs and LPS treatment may selectively increase extracellular ROS production from macrophages. Moreover, the intracellular ROS production in AML-12 cells were upregulated upon co-culture with AuNPs and LPS-treated murine peritoneal macrophages, but not by co-culture with identically treated HEK293 cells, which indicates the enhancement of ROS in hepatocytes by selectively interacting with treated macrophages (Fig. 7B). Next, AML-12 cells were directly treated with a macrophage culture medium conditioned with AuNPs and LPS, either alone separately or in combination. Intracellular ROS in AML-12 cells was only upregulated by conditioned medium from macrophages co-treated with AuNPs and LPS (Fig. 7C). Accordingly, apoptosis was markedly increased only after cells were cultured in AuNPs and LPS-co-conditioned macrophage medium (Fig. 7D). The requirement of concurrent treatment of macrophages by AuNPs and LPS were further shown by the results that ROS levels and apoptosis were not enhanced by the addition of LPS or AuNPs in the medium where co-treated macrophages were cultured or in a medium from any other combinational treatment without both AuNPs and LPS (Additional file 13: Fig. S11A, B). To address the specific role of extracellular ROS, the conditioned medium from AuNPs and LPS treated macrophages were added with the ROS scavenger NAC. In this case, both the increase in intracellular ROS production and apoptosis of AML-12 cells were abolished (Fig. 7D, E). Moreover, H_2_O_2_ was directly added to the culture medium of AML-12 cells, and H_2_O_2_ treatment also dose-dependently promoted intracellular ROS production and apoptosis in AML-12 cells (Fig. 7F, G). Taken together, these data suggest that the release of extracellular ROS requires AuNP and LPS co-treatment in macrophages and may mediate the crosstalk between macrophages and hepatocytes, which triggers ROS generation and apoptosis induction in hepatocytes.

### Extracellular ROS stimulates calcium-dependent NOX2 activation to upregulate ROS generation and induce apoptosis in hepatocytes

**Fig. 8:**
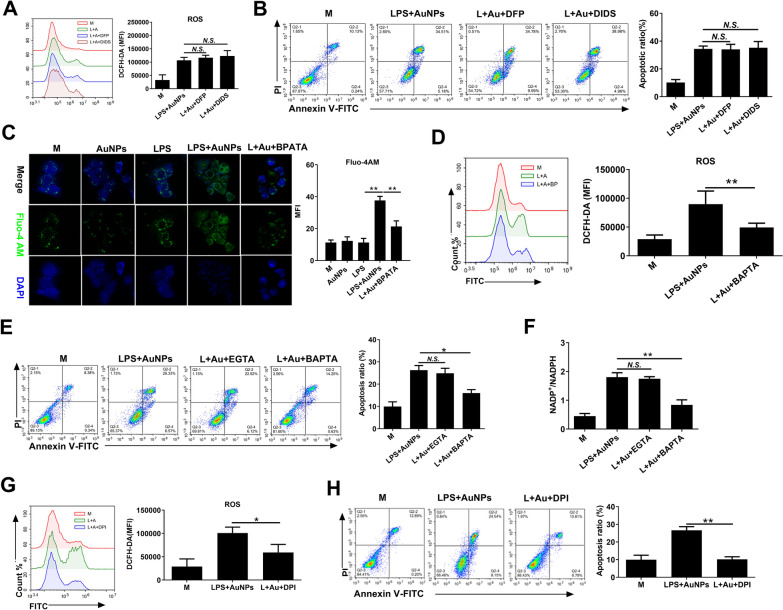
Extracellular ROS stimulates calcium-dependent NOX2 activation to upregulate ROS generation and induce apoptosis in hepatocytes. **A**, **B** AML-12 cells were treated with AuNPs and LPS-primed macrophage supernatants with or without DFP or DIDS. The production of ROS (**A**) and apoptosis (**B**) were detected via flow cytometry. **C**, **D** AML-12 cells were treated by primed macrophage supernatants with or without a calcium chelator BAPTA-AM. Intracellular calcium was quantified via Fluo-4AM staining (**C**) and ROS was measured by flow cytometry (**D**). **E**, **F** AML-12 cells were treated by primed macrophage supernatants with or without BAPTA-AM or EGTA. Apoptosis was measured by flow cytometry (**E**) and NOX2 activity was detected by measuring the NAD+ /NADH ratio (**F**). **G**, **H** AML-12 cells were treated by primed macrophage supernatants with or without DPI. ROS (**G**) and apoptosis (**H**) were measured via flow cytometry. Cell treatment was 4 h unless indicated (n = 3). N.S., no significance, *: P < 0.05, **: P < *0.01*

We next investigated the mechanism by which extracellular ROS upregulates intracellular ROS and promotes apoptosis in hepatocytes. Extracellular ROS can directly enter cells via transmembrane transport through aquaporins or chloride channels [24, 25]. However, both the increase in intracellular ROS (Fig. 8A) and the induction of apoptosis (Fig. 8B) in AML-12 cells induced by AuNPs and LPS-conditioned macrophage supernatants were not markedly suppressed by either an aquaporin 3 inhibitor (DFP00173) or a CLC3 inhibitor (DIDS), suggesting that extracellular ROS may not increase intracellular ROS in hepatocytes via direct transmembrane transport. On the other hand, extracellular ROS was previously observed to trigger ROS generation by inducing calcium-dependent NOX2 activation [26]. Here, we found that AuNPs and LPS-conditioned macrophage supernatants increased intracellular calcium levels in AML-12 cells, compared to no substantial calcium elevation upon treatment with culture medium conditioned with either agent alone (Fig. 8C). Notably, the intracellular calcium chelator BAPTA-AM diminished intracellular calcium level (Fig. 8C) and suppressed ROS elevation (Fig. 8D) in AML-12 cells in response to treatment with AuNPs and LPS-conditioned medium. BAPTA-AM also abolished the synergistic apoptotic effect of AuNPs and LPS-conditioned medium (Fig. 8E) or direct H_2_O_2_ treatment (Additional file 14: Fig. S12) in AML-12 cells. In contrast, treatment with the extracellular calcium chelator EGTA did not inhibit apoptosis in AML-12 cells, despite being cultured in a conditioned medium. Moreover, BAPTA-AM, instead of EGTA, inhibited the elevation of the NADP^+^/NADPH ratio in AML-12 cells after treatment with AuNPs and LPS-conditioned medium, further suggesting that extracellular ROS increases intracellular calcium concentrations to activate NOX2 in AML-12 cells (Fig. 8F). In addition, both ROS generation and apoptosis induction in AML-12 cells induced by AuNPs and LPS-conditioned medium were suppressed by the NOX2 inhibitor DPI (Fig. 8G, H). These results further suggest that extracellular ROS release by AuNPs and LPS-conditioned macrophages activates calcium-dependent NOX2 and induces apoptosis in hepatocytes.

### LPS increases AuNPs uptake in macrophages to augment the induction of apoptosis

**Fig. 9:**
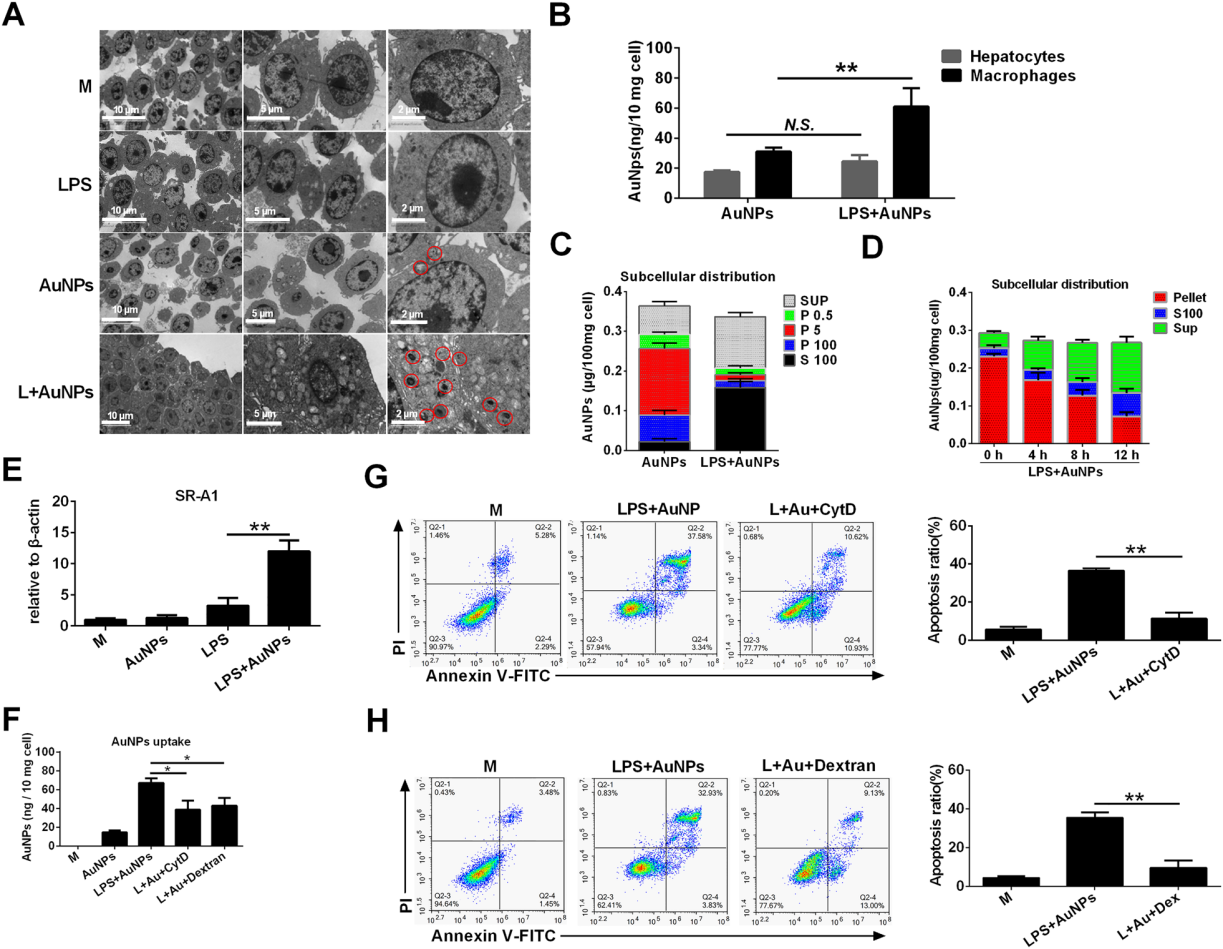
LPS increases the SRA-dependent AuNPs uptake in macrophages to mediate apoptosis induction. **A** Murine peritoneal macrophages were treated with AuNPs and LPS, separately or in combination, for 4 h. The intracellular distribution of AuNPs was visualized via TEM imaging (labeled by red circles). **B** Murine peritoneal macrophages or AML-12 cells were treated with AuNPs alone or in combination with LPS for 4 h. The content of Au per 10 mg cells was quantified via ICP-MS. **C**, **D** Macrophages were treated with AuNPs alone or together with LPS for 4 h. Cell homogenates were processed via gradient centrifugation to obtain the P0.5, P5, P100, S100, and Pellet fractions. The Au content in these fractions was detected via ICP-MS. **E** Macrophages were treated as in C and D. The mRNA expression of SR-A1 was detected via RT-PCR. **F** Macrophages were pre-treated with dextran sulfate or cytochalasin D for 2 h, followed by LPS and AuNP treatment for another 4 h. The Au content per 10 mg cells was quantified via ICP-MS (**G**, **H**) Cells were pre-treated with dextran sulfate (**G**) and cytochalasin D (**H**) for 2 h and then treated with LPS and AuNPs as indicated in C. Apoptosis was examined via Annexin V-FITC/PI staining. N.S., no significance, *: P < 0.05, **: P < *0.01*

Nanoparticles are recognized as xenobiotics by the host and are frequently endocytosed by phagocytes [27]. Therefore, we investigated whether LPS augmented AuNP uptake by macrophages and enhanced apoptosis induction. As shown via TCM, AuNPs were visualized as agglomerates with high electron densities and were mainly distributed in vesicular structures after treatment with cultured murine peritoneal macrophages. The agglomerates were not detected in control cells or cells treated with LPS alone. In addition, intracellular AuNP distribution was elevated upon co-treatment with LPS (Fig. 9A). The results of ICP-MS also confirmed that LPS co-treatment markedly increased the content of AuNPs in macrophages, compared to no significant upregulation of AuNPs content in identically treated hepatocytes (Fig. 9B). Subcellular fractions of macrophages were then prepared via differential centrifugation, and the content of AuNPs in each fraction was examined. AuNPs were mainly distributed in the P5 (mitochondria) and P100 (endoplasmic reticulum and Golgi apparatus) fractions when administered to macrophages alone. A profound decline of AuNPs content in the P5 and P100 fractions was detected after additional LPS co-treatment, accompanied by an increased AuNP accumulation in the S100 fraction (cytosolic components) (Fig. 9C). Time-lapse analysis also demonstrated that AuNPs were initially distributed in the pellet fraction (membrane-bound organelles) but were markedly transported into the S100 fraction (cytosolic components) 6 h after exposure to AuNPs and LPS, demonstrating a dynamic intracellular translocation of AuNPs after macrophage endocytosis (Fig. 9D). Next, we examined whether the increase in endocytosis was responsible for the pro-apoptotic effects of AuNPs. Scavenger receptor A1 (SR-A1) acts as an important phagocytic receptor for the recognition and uptake of AuNPs [28]. Co-treatment with AuNPs and LPS significantly increased the mRNA expression of SR-A1 in macrophages compared to LPS treatment alone, although AuNPs alone did not upregulate SR-A1 expression (Fig. 9E). Then, using ICP-MS, we demonstrated that the increased AuNP uptake due to combined AuNPs and LPS treatment was disrupted by an endocytosis inhibitor, cytochalasin D, or an SR-A1 antagonist, dextran (Fig. 9F). Moreover, apoptosis in macrophages was significantly inhibited upon treatment with cytochalasin D (Fig. 9G) and dextran (Fig. 9H). Therefore, these data indicate that LPS enhances the SRA-dependent uptake of AuNPs to increase apoptosis induction in macrophages.

## Discussion

AuNPs are generally considered to be biocompatible, whereas they may become toxic or affect the toxicity of other molecules upon interaction within chemical or biological systems. Given that AuNPs are increasingly applied in infectious or inflammatory conditions, this study examined whether AuNPs play harmful roles in these situations by using a typical bacterial LPS challenge model. We found that acute exposure to 10 nm AuNPs significantly increased the lethal hepatotoxic effect of LPS challenge, along with an increased hepatic accumulation of AuNPs in the presence of LPS. Interestingly, a recent study reported that AuNPs synergized with polysorbate-20 to induce toxicity in embryonic zebrafish [7]. The authors suggested the need to consider the potential mixture toxicity in particle manufacturing. Our study describes a previously unknown adverse effect of AuNPs that may sensitize a host to the immunotoxic activity of bacterial components and exacerbate liver injury, thereby implicating the potential risks of using biocompatible nanoparticles in infectious or inflammatory settings.

The inflammatory activity of LPS is critical for triggering ALI, while immune reactions, especially inflammation, are also often regarded as a major biological index for the safety evaluation of nanoparticles [29, 30]. Nevertheless, the contamination of NP surfaces by LPS, which is not routinely examined in scientific research, may result in the misinterpretation of results concerning the inflammatory potential of nanomaterials [4, 30, 31]. In this study, we excluded potential LPS contamination in each AuNP suspension, yet we were unable to detect any direct pro-inflammatory effect. Our results are consistent with previous studies which reported that treatment with non-conjugated AuNPs with sizes at 10 or 60 nm did not cause cytotoxicity or the production of pro-inflammatory cytokines in macrophages [32, 33]. Moreover, we further demonstrated that AuNPs did not increase the inflammatory ability of LPS by employing both ELISA and westernblot methods to evaluate the expression of inflammation associated proteins. Therefore, the augmentation of hepatotoxic effects by AuNP exposure may be attributed to mechanisms other than amplifying systemic or local inflammation.

The massive loss of hepatic parenchymal cells due to apoptosis is another fundamental paradigm that characterizes hepatic injury induced by LPS or other hepatotoxic insults [34, 35]. In our study, hepatic apoptosis was not upregulated by treatment with AuNPs alone but was significantly induced upon co-injection with AuNPs and LPS. A recent study demonstrated that AuNPs could synergize with antibiotics to enhance apoptosis-like death in *Salmonella* species, which similarly reveals the capacity of AuNPs to enhance apoptosis by functionally synergizing with other substances [36]. LPS can trigger apoptosis through both extrinsic and intrinsic pathways [37]. Our results demonstrated that co-treatment with AuNPs and LPS activated the mediators involved in intrinsic apoptosis. These findings are in accordance with observations that treatment with AuNPs did not enhance inflammatory responses, which are key to the induction of extrinsic apoptosis [38]. In addition, the feasibility of cellular contact and uptake of 10 nm AuNPs may also explain the phenomenon that AuNPs can favorably induce intrinsic apoptosis, which is routinely initiated within cells [39]. Moreover, we found that inhibition of apoptosis significantly alleviated liver injury and improved survival in AuNP- and LPS-challenged mice, thus further revealing the possible association between the induction of hepatic apoptosis and the occurrence of liver injury by co-treatment with AuNPs and LPS.

Hepatocytes and hepatic macrophages are primary hepatic cells which are responsible for the ultimate clearance of foreign substances, including nanoparticles [40]. Indeed, nanoparticles that enter hepatocytes can be removed via the hepatobiliary pathway, while non-parenchymal cells (e.g., hepatic macrophages) are the typical phagocytes responsible for the uptake of nanoparticles and the induction of cellular responses, including inflammation, oxidative stress, and cell death [41]. Thus, it is not surprising that these insults cause their preferential target cells to be damaged and undergo cellular apoptosis. Here, we demonstrated that apoptosis and deformity were upregulated in both hepatocytes and hepatic macrophages in mice that received an AuNPs and LPS co-injection. The immunofluorescence experiment was designed in our study to identify the distribution of apoptosis (TUNEL staining) in hepatocytes (cytokerin-18 staining) or hepatic macrophages (F4/80 staining) in murine liver sections. As TUNEL staining occurs in nucleus while cytokerin-18 and F4/80 are membrane proteins. Therefore, it is reasonable that their overlaps are low due to spatial distances between nucleus and cytomembrane, whereas TUNEL and DAPI staining which are both distributed in the nucleus were thus highly colocalized. Notably, the elevation of apoptosis by AuNPs and LPS co-treatment was only detected in monocultured macrophages, but not in monocultured hepatocytes. Co-culture experiments further confirmed the requirement of intercellular crosstalk in mediating apoptosis in vitro and macrophage depletion approaches in vivo. In fact, it has been demonstrated that hepatocyte apoptosis can be triggered via crosstalk with hepatic macrophages, as shown in LPS-elicited murine hepatitis or in non-alcoholic steatohepatitis [42, 43]. Our study may indicate a new type of intercellular crosstalk between hepatic macrophages and hepatocytes that induces apoptosis in both cell types. Notably, this effect was observed in both murine and human hepatocytes but not in control parenchymal cells, suggesting a universal but selective interacting mechanism between macrophages and hepatocytes among species. In addition, our results may also offer additional explanations about the underlying mechanisms of liver injury caused by exposure to foreign substances.

Elevated intracellular ROS generation, which is a key biological effect of nanoparticles, can damage intracellular components and promote apoptosis [44]. However, a recent study demonstrated that 25 nm AuNPs produced low-level oxidative stress when treated at sub-cytotoxic concentrations (1–50 μM) in J774A.1 cells or primary human macrophages [45]. We used similar sizes and doses of AuNPs and consistently found that AuNPs alone did not substantially enhance ROS generation in macrophages. In contrast, ROS generation was markedly upregulated when AuNPs were co-treated with LPS in macrophages, accompanied by a disruption in redox balance into a pro-oxidative status. Together with data showing that the ROS scavenger GSH reduced apoptosis and ameliorated liver injury upon AuNP and LPS administration, these results not only confirm our observations in this study that AuNPs amplified the toxicity of LPS but may also reveal a central role of ROS production in mediating the augmentation of hepatic apoptosis and injury. Although it has been extensively demonstrated that nanoparticles induce ROS generation, it remains uncertain whether they activate NOX2 or enhance mtROS generation. Using an RNA-seq approach, we obtained transcriptional profiles that identified a marked upregulation of NOX2-associated transcripts. Moreover, we demonstrated that the concurrent exposure to AuNPs and LPS selectively induced NOX2 activation to increase ROS production. To the best of our knowledge, this study was the first to report the effect of administering AuNPs in amplifying NOX2 activation induced by LPS. Notably, a recent study also reported the use of curcumin-conjugated poly (lactic-co-glycolic acid) nanoparticles to enhance NOX2/gp91phox expression and ROS production in neutrophils [46]. Their findings were in line with our results and confirmed the possibility that nanoparticles upregulate ROS generation by activating NOX2.

Most previous studies have reported that hepatic macrophages induce hepatotoxicity by upregulating cytokine production and that crosstalk between hepatic macrophages and hepatocytes is commonly mediated by the release of cytokines, including TNF-α, IL-6, and IL-1β [47, 48]. In our study, AuNPs did not promote cytokine release from LPS-stimulated macrophages, thereby excluding the relevance of this mechanism. ROS can travel across the cell membrane into the extracellular space or become produced outside cells via the membrane NOX complex, where they serve as important regulators of intercellular communications [49–51]. Indeed, recent studies demonstrated that the elevated production of ROS outside cells plays unique roles in apoptosis induced by cold atmospheric helium plasma [52] or reducing the proliferation and antitumor function of T cells [53]. Here, we further demonstrated that AuNPs and LPS increased extracellular ROS levels to mediate the crosstalk between hepatic macrophages and hepatocyte. An increasing amount of evidence has revealed that extracellular ROS affects the cellular state in a multifaceted manner, such as the modulation of Ca^2+^ signaling [54], activation of the autophagy machinery, and an increase in intracellular ROS levels [55]. Moreover, extracellular ROS can increase intracellular ROS levels via transmembrane transport [25] or induce calcium-dependent NOX2 activation [56]. Our results did not indicate transmembrane transport as the major route for the increase in intracellular ROS levels. Instead, we observed elevated calcium levels in cultured hepatocytes stimulated with ROS, which subsequently induced NOX2 activation. Moreover, both ROS generation and apoptosis in hepatocytes induced by extracellular ROS stimulation were suppressed through calcium chelation and treatment with a NOX2 inhibitor. Based on these data, our study may define a new role by which extracellular ROS function as signaling molecules that connect hepatic macrophages with hepatocytes in generating intracellular ROS, thereby inducing apoptosis and liver injury.

Multiple studies have documented the uptake of nanoparticles by macrophages and their trafficking via a common endosomal-lysosomal pathway [57]. We demonstrated here that the uptake of AuNPs was enhanced by LPS co-treatment in macrophages but not in hepatocytes. In addition, the internalized AuNPs initially appeared within membrane-bound organelles and then entered the cytosol. A recent study reported that cytosolic PCNA interacts with p47phox and controls NADPH oxidase-induced NOX2 activation in neutrophils [58]. Therefore, we believe that the transfer of AuNPs into the cytosol may also increase the opportunity for AuNPs to interact with NOX2 and regulate ROS production. A previous study suggested that SRA-dependent endocytosis, not macropinocytosis, plays a major role in the uptake of gold colloids in macrophages without inducing a pro-inflammatory response [59]. Since we also ruled out inflammation induction due to AuNP entry, we speculated that AuNPs might also be internalized by SRA and then co-operated with LPS in macrophages. As expected, SR-A1 was upregulated by AuNPs, and the chemical antagonist of SRA suppressed the effect of AuNPs treatment. Although these results did not prove to exclude the involvement of other endocytic pathways, they may reveal that SRA-dependent endocytosis is required, at least in part, to mediate AuNP uptake and the induction of apoptosis in macrophages.

## Conclusions

In summary, our results demonstrate that acute exposure to 10 nm unmodified AuNPs exacerbates liver injury and increases mortality in mice challenged with bacterial LPS. AuNPs do not promote systemic or hepatic inflammation; instead, they are increasingly internalized by hepatic macrophages upon co-treatment with LPS to synergistically upregulate NOX2-dependent cytosol ROS generation and induce apoptosis in macrophages. Concurrent AuNPs and LPS exposure also induces extracellular ROS generation by macrophages to mediate intercellular crosstalk, thereby promoting apoptosis in hepatocytes and aggravating liver injury. Our study may provide a new insight into the immunotoxic effect of AuNPs to aggravate hepatotoxicity by increasing cellular sensitivity to LPS and inducing intercellular crosstalk in hepatic cells.

## Materials and methods

### Nanoparticle preparation

AuNPs (10 nm) were purchased from XFNANO Materials Tech Co., Ltd. (Nanjing, China). The morphology of the AuNPs was examined via TEM (JEOL JEM-1400, Japan), and the primary size of the AuNPs was determined by measuring at least 50 randomly selected particles using ImageJ software. The absorption spectra were obtained via spectral scanning at wavelengths of 400–900 nm using a microplate reader (Thermo Varioskan® Flash, USA). The hydrodynamic size and zeta potential of AuNPs dispersed in ultrapure water, PBS, or DMEM solutions (Gibco, USA) were determined using a Zetasizer Nano ZSP (Malvern, UK).

### Animals and treatment

Wild-type BALB/c and C57/BL6 mice (male, 8–10 weeks) were purchased from HFK Bioscience Co., Ltd. (Beijing, China). NOX2-mutant C57/BL6 mice were obtained from Nanjing University. All mice received humane care, and all animal experiments were performed in accordance with national guidelines under a protocol approved by the Animal Ethics Committee of the Army Military Medical University.

BALB/c mice were administered AuNPs alone or 30 min after *Escherichia coli* O55:B5 LPS (Sigma) challenge. The doses used were 20 and 10 mg/kg for AuNPs and LPS, respectively, unless indicated otherwise. In some experiments, mice were pre-injected with 10 mg/kg clodronate liposomes (FormuMax, USA), 10 μg/kg Z-DEVD-FMK (MCE, USA), 5 mg/kg Z-VAD (OMe)-FMK (MCE), 10 mg/kg GSH (Sigma), or 50 μg/kg DPI (Sigma) for 30 min before being challenged with LPS and AuNPs. All injections were administered intraperitoneally. Survival was observed for 72 h. Afterward, the mice were anesthetized and sacrificed 6 h after injection for sample collection. Tissue sections were prepared for hematoxylin and eosin (H&E) and immunofluorescence staining. Serum or tissue homogenates were prepared for the measurement of cytokines, enzymes, or other protein markers.

### Cell culture and treatment

Primary peritoneal macrophages were isolated from wild-type or NOX-mutant mice, as described previously [60]. The human embryonic kidney (HEK)-293 cell line and the alpha mouse liver 12 (AML-12) cell line were purchased from ATCC (USA). Human umbilical vein endothelial cells (HMVECs) and the mouse fibroblast cell line L929 were kindly provided by Dr. Yingjuan Mei from the Army Military Medical University. AML-12 or LO2 cells were cultured in Dulbecco’s Modified Eagle Medium/nutrient mixture F-12 (DMEM-12) (GIBCO) containing 1% ITS Liquid Media Supplement (Sigma), 10% fetal bovine serum (FBS) (Hyclone, USA), and 40 ng/ml dexamethasone (Sigma). Other cells were cultured in DMEM containing 10% FBS. All cells were incubated at 37 °C in a humidified incubator supplemented with 5% CO_2_.

Cells were treated with 10 ng/ml *E. coli* O55:B5 LPS alone or further with AuNPs (0, 10, and 20 μg/ml) 30 min later. In some experiments, cells were pre-treated with 20 μM Z-DEVD-FMK, 100 μM Z-VAD (OMe)-FMK, 5 mM glutathione (GSH), 10 mM N-acetyl-L-cysteine (NAC), 100 mM mito-TEMPO, 0.3 μg/ml dextran sulfate, 0.3 μg/ml cytochalasin D and 10 mM DPI for 30 min or 2 h and then subjected to AuNPs and LPS treatment. In co-culture experiments, cell monolayers were separately inoculated into the upper and lower compartments of a Transwell chamber (Corning, China) with a pore diameter of 3 μm before being treated with AuNPs and LPS. In some experiments, after treating AML-12 cells with H_2_O_2_, ROS levels and apoptosis were directly measured. In some laboratories, cell supernatants treated with AuNPs and LPS separately or in combination were obtained via co-cultivation, and AML-12 (with 0.4 μM DFP00173, 200 μM DIDS, 50 μM EGTA, and 10 μM BAPTA-AM pretreatment for 30 min) cells were cultured using the supernatant mentioned above to determine levels of ROS and apoptosis.

### Biological measurement

The enzymatic activities of ALT, AST, caspase-3, caspase-9, MIDA, MPO, SOD, and CAT in the serum and tissues were quantified using commercial biochemical kits (Nanjing Jiancheng Bioengineering Institute, China) according to the manufacturer’s instructions. Tissue enzyme activity was normalized to the protein content.

### Quantification of AuNPs by ICP-MS

The concentration of AuNPs in cells or tissues was quantified using icapQ ICP-MS (Thermo Scientific, USA). Briefly, cell lysates or tissue homogenates were lyophilized and digested with aqua regia (A mixture of concentrated hydrochloric acid and concentrated nitric acid in a volume ratio of 3:1). After complete digestion, the solution was adjusted with ultrapure water to obtain a final hydrochloric acid concentration of 2% v/v. The resulting solution was filtered through a membrane filter (pore size = 0.45 μm) to remove the precipitates. Total Au content was analyzed using ICP-MS. Au internalization in cells or Au distribution in mice was determined by calculating the amount of Au per 10 mg and 100 mg of cells in dry weight or per g of tissue in dry weight.

### Histological examination of liver tissue sections

Liver tissues were embedded in 4% polyformaldehyde, and 5-µm tissue sections were prepared and stained with H&E. The histological morphology of the liver sections was observed using an upright microscope (ZEISS, Germany).

### Immunofluorescence analysis

Frozen sections of liver tissue were prepared immediately after sampling. The sections were fixed with ice-cold acetone for 15 min, sealed with 10% goat serum at 4 °C for 30 min, and permeabilized with 0.2% Triton X-100 for 15 min. This was followed by incubation of the sections with antibodies against cytokeratin 18 (1:100, Abcam, USA) or F4/80 (1:250, CST, USA) at 4 °C overnight. The sections were incubated with Cy3-conjugated secondary antibody (Beyotime, 1:400, China) and dyeing with TUNEL and DAPI (Beyotime, China). Changes in cellular calcium ion concentration were analyzed via immunofluorescence staining. Briefly, after macrophages were treated with LPS and AuNPs, the cells were incubated with Fura-4 AM (MCE, USA) and counterstained with DAPI (Beyotime). Fluorescence images were captured using a ZEISS 780 laser confocal microscope (Zeiss, Germany).

### Apoptosis detection

Apoptosis was detected through the terminal deoxynucleotidyl transferase-dUTP nick-end labeling (TUNEL) assay or via PI/annexin V staining. For the TUNEL assay, frozen liver sections were permeabilized and incubated with the TUNEL test solution (Beyotime) for 60 min. Fluorescence was captured using a laser confocal microscope. For PI/annexin V staining, cells were resuspended in 100 μl of binding buffer containing 5 μl of PI and 10 μl of annexin V solution (BD Biosciences, USA) and incubated at 25℃ for 15 min. The number of apoptotic cells was measured via flow cytometry (ACEA NovoCyte, USA).

### ROS detection

Cells were preloaded with the cytosol ROS indicator DCFH-DA (10 μM, Sigma) or the mitochondrial ROS (mtROS) indicator MitoSOX Red (2.5 μM, Molecular Probes, USA) for 15 min and then treated with AuNPs and LPS for 4 h. A liquid ROS detection kit (BestBio, China) was used to supplement ROS in the cell culture supernatant [61]. Cytosolic ROS and mtROS levels were measured using an ACEA NovoCyte flow cytometer system (San Diego, CA, USA).

### NOX2 activity assay

NOX2 activity was quantified by measuring the ratio of NADP + /NADPH using an NADP/NADPH quantization kit (Sigma) according to the manufacturer’s instructions.

### RNA Sequencing (RNA-seq)

Total RNA was extracted using a Total RNA Extractor kit (Sangon, China). RNase-free DNase was used to remove contaminating DNA before RNA-seq was performed by Bio-Engineering Co., Ltd. (Shanghai, China). The values in the heat map represent the z-scores calculated for NOX, mtROS, and antioxidant-related genes in the control group or in the groups treated with LPS and AuNPs separately or in combination.

### Intracellular AuNPs imaging

Intracellular AuNPs were visualized using TEM. Briefly, the cells were collected and fixed in 2.5% glutaraldehyde solution overnight. Then, 70 nm ultra-thin cell sections were obtained. Bio-TEM observations were made according to the standard sample preparation procedures for bio-TEM (JEOL JEM-1400, Japan).

### Analysis of the subcellular distribution of AuNPs

The subcellular distribution of AuNPs was detected via ultracentrifugation, as described previously [62]. Briefly, cell homogenates were centrifuged at 100,000×*g* for 60 min to separate cytosolic extracts (S100) and pellets (P100) or were centrifuged at 500×*g* for 15 min, 5000×*g* for 30 min, and 100,000×*g* for 60 min to obtain the P0.5, P5, P100, and S100 cellular fractions, respectively. After separation, the Au content was measured via ICP-MS.

### RT-PCR

Total RNA was extracted from the harvested peritoneal macrophages using a TRIzol kit (Tiangen Biotech, China) and was reverse-transcribed into single-strand cDNA using a ReverTra Ace-α kit (TOYOBO, Japan). The cDNA template was mixed with a SYBR Green PCR Mastermix (TOYOBO) and the corresponding primers (Additional file 15: Table S1) for quantitative real-time PCR, which was performed on a CFX96™ thermal cycler (Bio-Rad). The expression levels of mRNA were normalized to the expression of the housekeeping gene actin.

### Western blot analysis

The treated cells were then lysed with T-PER protein extraction reagent containing protease cocktail and phosphatase inhibitor (Roche, Basel, Switzerland), or put animal tissue in the lysate, homogenize, and proteins were separated by SDS-PAGE and transferred onto PVDF membranes. Blots of protein were incubated with primary antibodies (1:1000 dilution) for cleaved caspase-2, cleaved caspase-3, TLR-4, TNF-α, IL-6, ERK1/2, p-ERK1/2, and Actin (All antibodies involved were purchased from (CST,USA) at 4 °C overnight. Blots were then incubated with HRP-conjugated secondary IgG antibodies (1:2000 dilution) at 37 °C for 1 h. Chemiluminescence images were developed with a SuperSignal Sensitivity Substrate kit and detected using a ChemiDoc XRS + imaging system (Bio-Rad).

### Statistics

Quantitative data are expressed as the mean ± standard deviation (SD). Statistical analysis was performed using GraphPad Prism 6.0 using one-way ANOVA and *Bonferroni corrections* for multiple comparisons. Statistical significance was set at *P* < 0.05.

## Supplementary Information

**Additional file 1: Fig. S1.** AuNPs were dispersed at a concentration of 10 μg/ml.

**Additional file 2: Table S2.** Physicochemical properties of AuNPs (10 μg/ml).

**Additional file 3: Fig. S2.** The UV–vis absorption spectrum of AuNPs (1 and 50 μg/ml) was measured by spectral scanning.

**Additional file 4: Table S3.** Physicochemical properties of AuNPs (1 and 50 μg/ml).

**Additional file 5: Fig. S3.** Evaluation of LPS contamination or inflammatory induction by individual AuNPs.

**Additional file 6: Fig. S4.** The expression of inflammatory factors in the liver of mice was treated with AuNPs and LPS alone or together.

**Additional file 7: Fig. S5.** Apoptosis in kidney and lung tissues by co-treatment of AuNPs and LPS.

**Additional file 8: Fig. S6.** Co-localization analysis of immunofluorescence images.

**Additional file 9: Fig. S7.** Apoptosis in other parenchymal or non-parenchymal cells induced by AuNPs and LPS co-treatment.

**Additional file 10: Fig. S8.** Apoptosis in co-cultured cells treated by AuNPs and LPS.

**Additional file 11: Fig. S9.** Identification of NOX2 deficient mice.

**Additional file 12: Fig. S10.** ROS determination of AuNPs, LPS and LPS + AuNPs in DMEM.

**Additional file 13: Fig. S11.** Effects of LPS and AuNPS alone or co-cultured supernatant on ROS and apoptosis of AML-12 cells.

**Additional file 14: Fig. S12.** The effect of BPATA on cell apoptosis caused by hydrogen peroxide.

**Additional file 15: Table S1.** Real-time PCR primers.

## Data Availability

All data generated or analysed during this study are included in this published article [and its Additional files].

## Abbreviations

AuNPs: Gold nanoparticles
ALI: Liver injury
ALT: Alanine transaminase
AST: Aspartate transaminase
ATCC: American type culture collection
CytD: Cytochalasin D
CAT: Catalase
CLL: Clodronate Liposomes
DCFH-DA: Dye 2',7'-Dichlorodihydrofluorescein diacetate
DMEM: Dulbecco's Modified Eagle Medium
DLS: Dynamic light scattering
DPI: Diphenyleneiodonium chloride
GalN: D-galactosamine
GABPB2: GA Binding Protein Transcription Factor Subunit Beta 2
GSH: Scavenger glutathione
HEK293 cells: Human embryonic kidney 293 cells
HMVECs: Human microvascular endothelial cells
ICP-MS: Inductively coupled plasma mass spectrometry
IL-6: Interleukin 6
LAL: Limulus amoebocyte lysate
LPS: Lipopolysaccharide
MIDA: Malondialdehyde
MPO: Myeloperoxidase
NADP/NADPH: Nicotinamide adenine dinucleotide phosphate/Reduced nicotinamide adenine dinucleotide phosphate
NOX2: Nicotinamide adenine dinucleotide phosphate (NADPH) oxidase 2
PBS: Phosphate buffer solution
ROS: Reactive oxygen species
RNA-seq: RNA Sequencing assay
SCO2: SCO Cytochrome C Oxidase Assembly Protein 2
SRA: A scavenger receptor
SOD: Superoxide dismutase
TEM: Transmission electron microscopy
TNF-α: Tumor necrosis factor alpha
TUNEL: Terminal deoxynucleotidyl transferase dUTP nick end labeling
DIDS: Disodium 4,4'-Diisothiocyanato -2,2'-stilbenedisulfonate Hydrate
BAPTA-AM: 1,2-Bis (2-aminophenoxy) ethane-N,N,N′,N′-tetraacetic acid tetrakis (acetoxymethyl ester)
EGTA: Ethylenebis (oxyethylenenitrilo) tetraacetic acid
