# Correction: Acute exposure to gold nanoparticles aggravates lipopolysaccharide-induced liver injury by amplifying apoptosis via ROS-mediated macrophage-hepatocyte crosstalk

**DOI:** 10.1186/s12951-023-01972-6

**Published:** 2023-07-26

**Authors:** Yongjun Yang, Shijun Fan, Qian Chen, Yongling Lu, Yuanfeng Zhu, Xiaoli Chen, Lin Xia, Qianying Huang, Jiang Zheng, Xin Liu

**Affiliations:** Medical Research Center, Southwest Hospital, Army Military Medical University, Chongqing, 400038 China

**Correction: Journal of Nanobiotechnology (2022) 20:37** 10.1186/s12951-021-01203-w

Following publication of the original article [1], the authors identified an error in Fig.9. The correct Fig. 9 is given below. The authors apologize for not noticing these errors prior to publication, and for any inconvenience caused. The original article has been corrected.

**Fig. 9:**
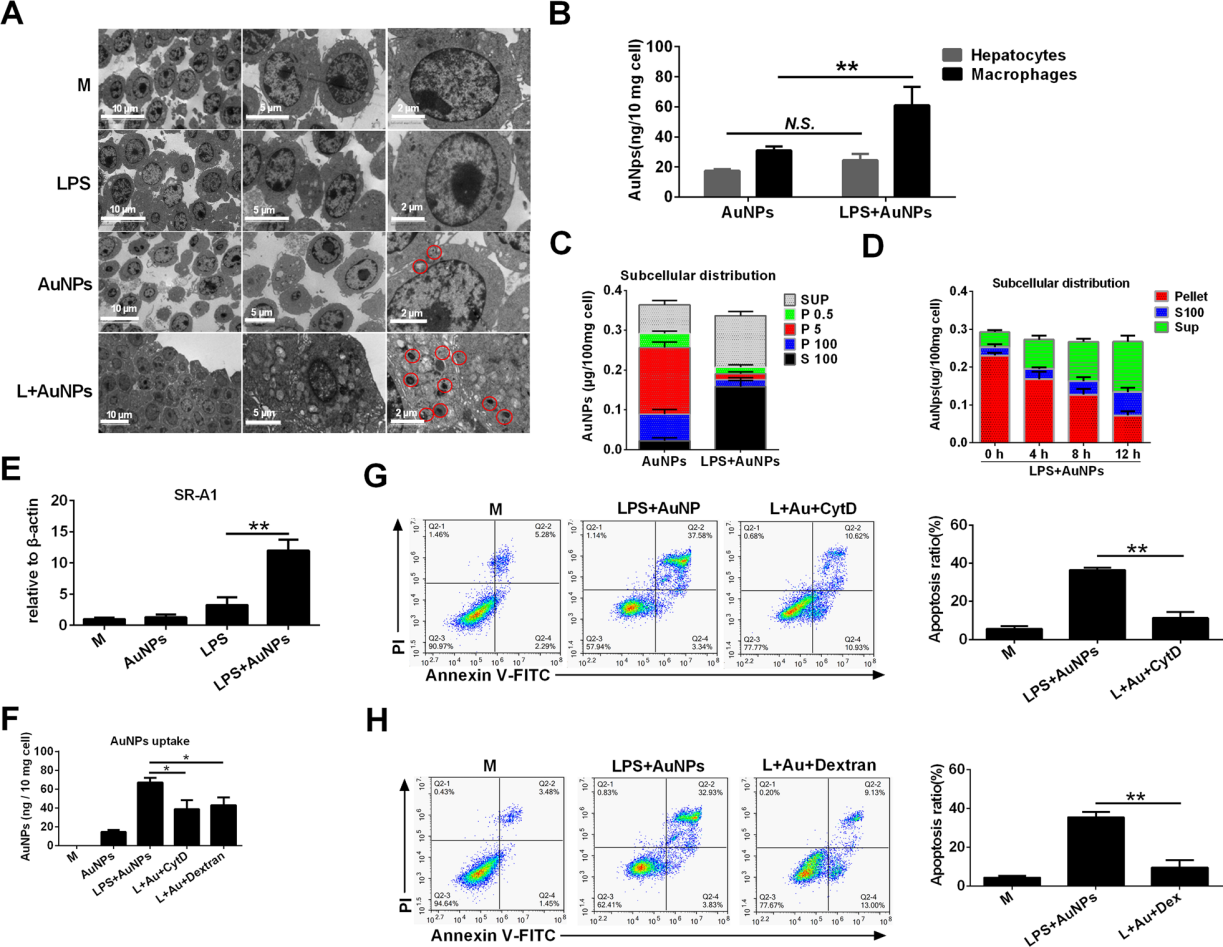
LPS increases the SRA-dependent AuNPs uptake in macrophages to mediate apoptosis induction. **A** Murine peritoneal macrophages were treated with AuNPs and LPS, separately or in combination, for 4 h. The intracellular distribution of AuNPs was visualized via TEM imaging (labeled by red circles). **B** Murine peritoneal macrophages or AML-12 cells were treated with AuNPs alone or in combination with LPS for 4 h. The content of Au per 10 mg cells was quantified via ICP-MS. **C**, **D** Macrophages were treated with AuNPs alone or together with LPS for 4 h. Cell homogenates were processed via gradient centrifugation to obtain the P0.5, P5, P100, S100, and Pellet fractions. The Au content in these fractions was detected via ICP-MS. **E** Macrophages were treated as in **C** and **D**. The mRNA expression of SR-A1 was detected via RT-PCR. **F** Macrophages were pre-treated with dextran sulfate or cytochalasin D for 2 h, followed by LPS and AuNP treatment for another 4 h. The Au content per 10 mg cells was quantified via ICP-MS. **G**, **H** Cells were pre-treated with dextran sulfate **G**) and cytochalasin D **H** for 2 h and then treated with LPS and AuNPs as indicated in **C**. Apoptosis was examined via Annexin V-FITC/PI staining. N.S., no significance, *: P < 0.05, **: P < 0.01
