# Supplementary figures and images for: Acute exposure to gold nanoparticles aggravates lipopolysaccharide-induced liver injury by amplifying apoptosis via ROS-mediated macrophage-hepatocyte crosstalk
Source: J Nanobiotechnology. 2022 Jan 20;20:37. doi: 10.1186/s12951-021-01203-w (PMC8772144; doi:10.1186/s12951-021-01203-w)

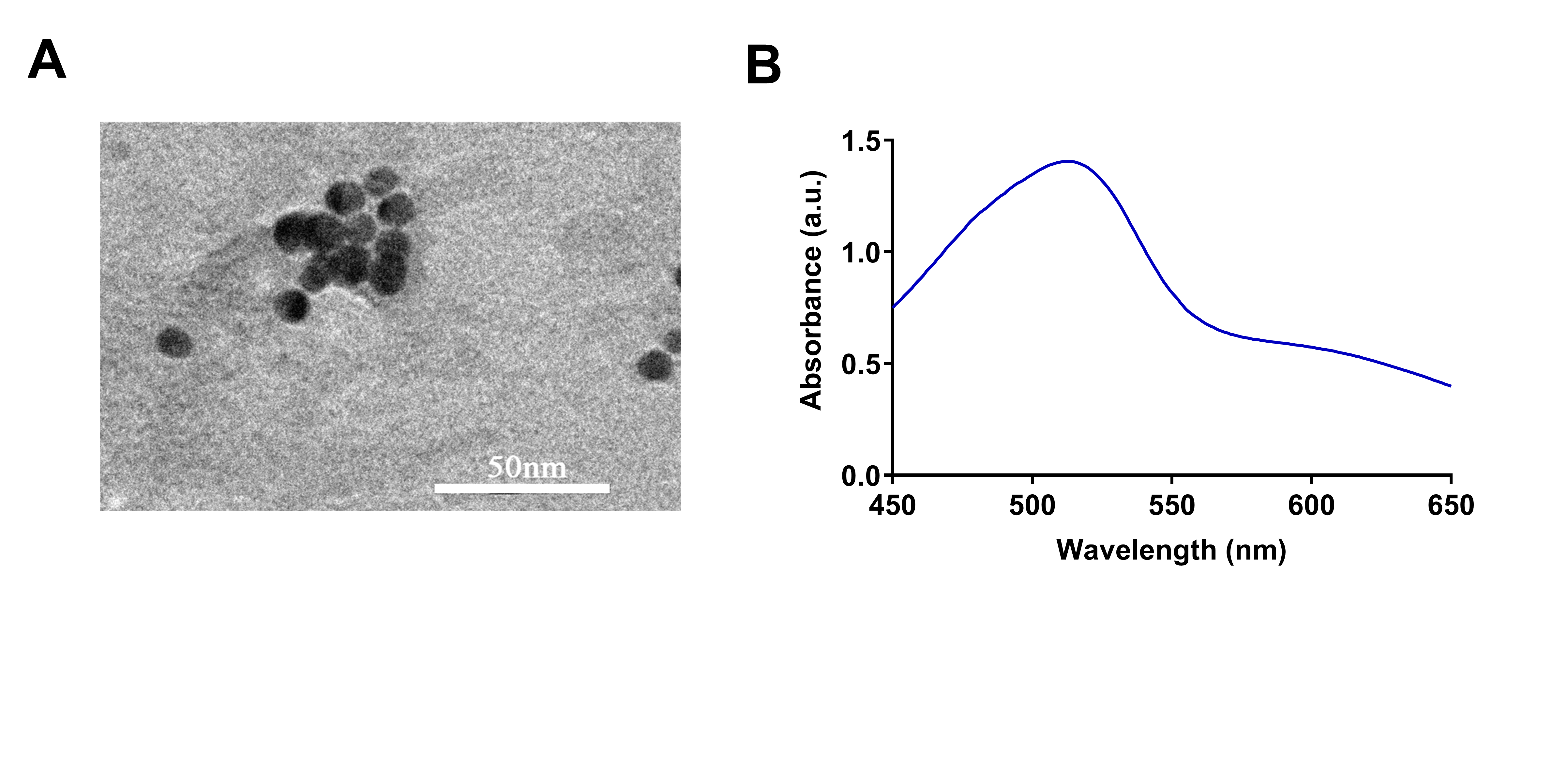

Supplement: Supplementary file 1 — Additional file 1: Fig. S1. AuNPs were dispersed at a concentration of 10 μg/ml. [file 12951_2021_1203_MOESM1_ESM.tif]

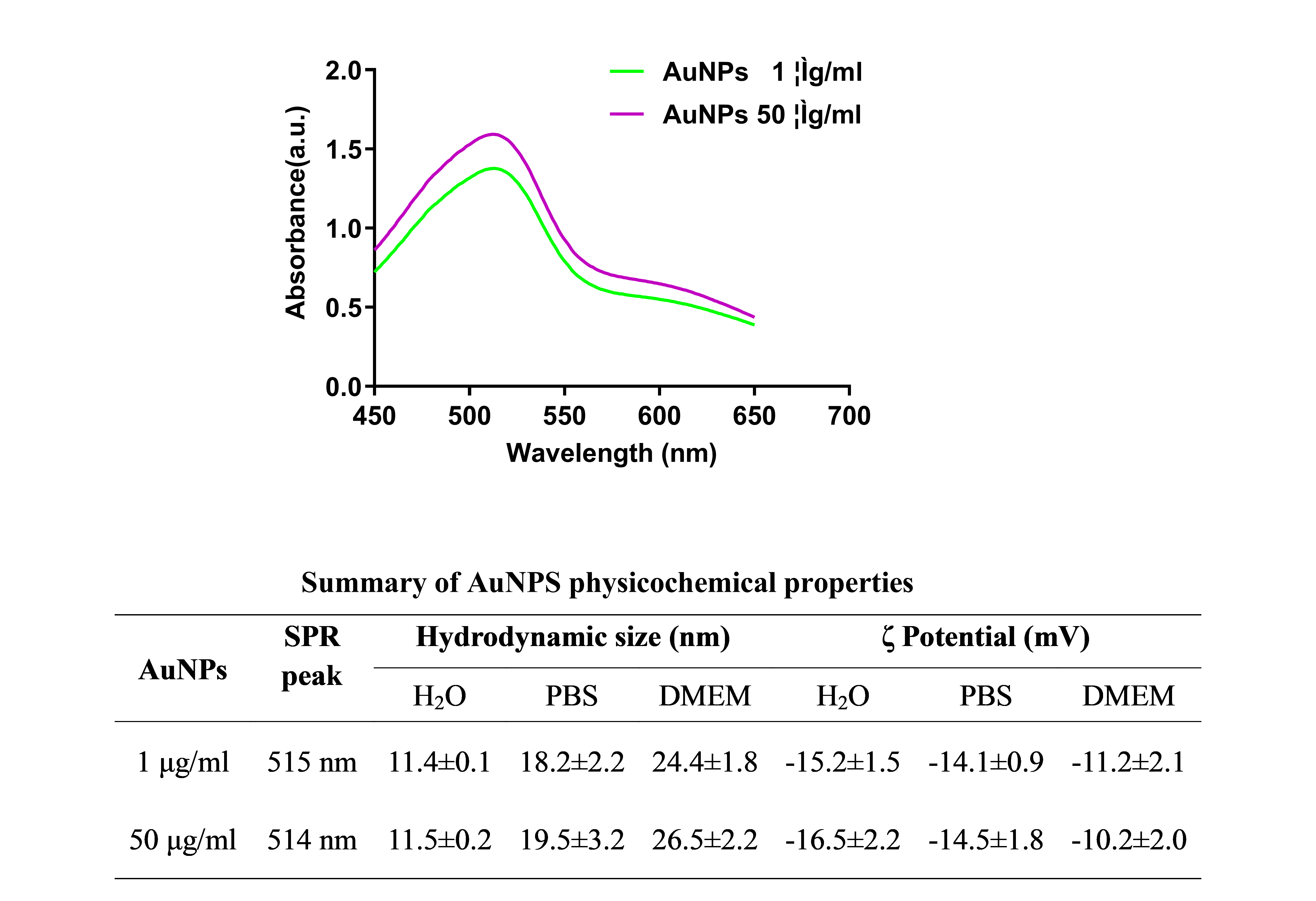

Supplement: Supplementary file 3 — Additional file 3: Fig. S2. The UV–vis absorption spectrum of AuNPs (1 and 50 μg/ml) was measured by spectral scanning. [file 12951_2021_1203_MOESM3_ESM.tif]

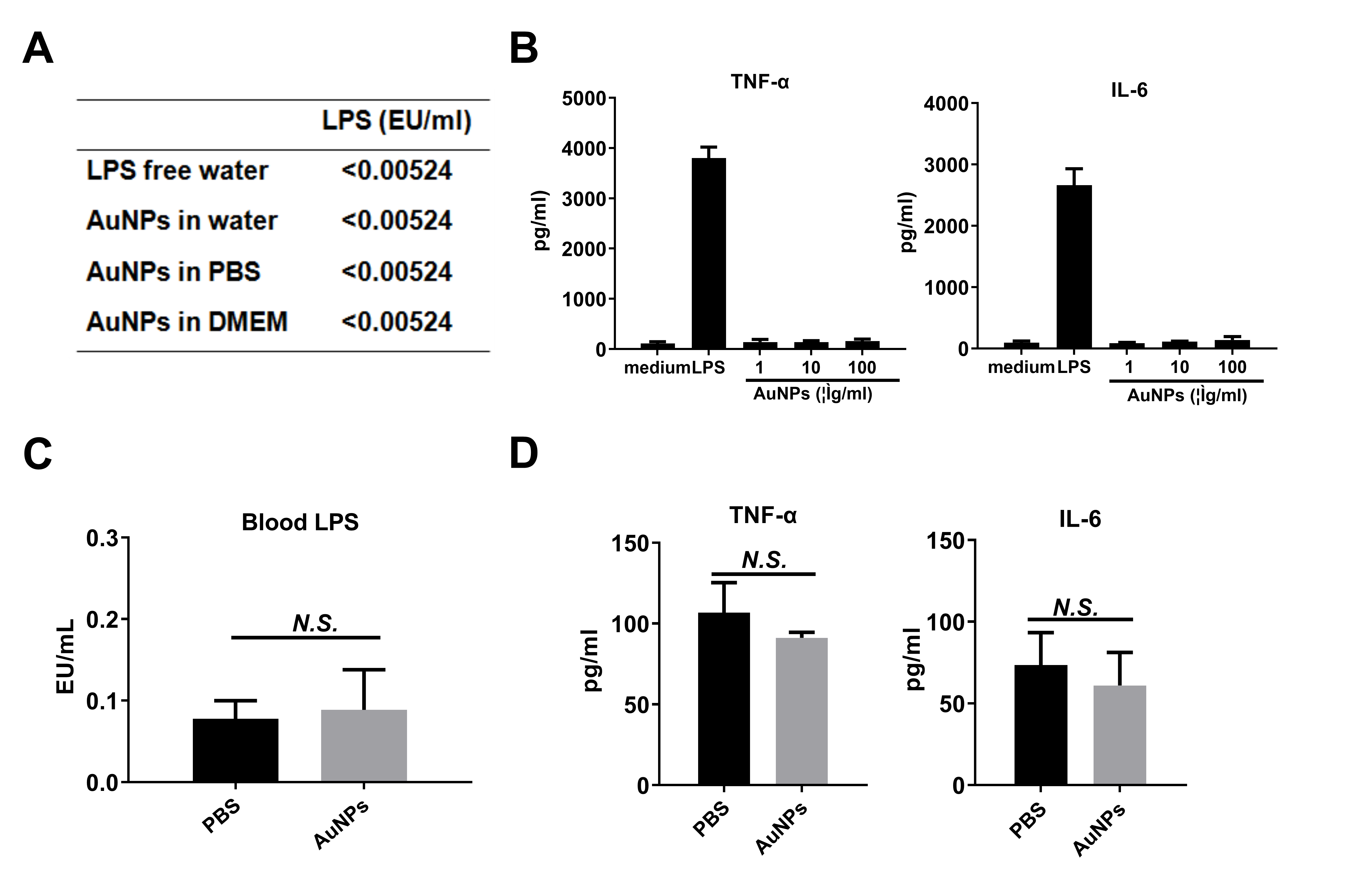

Supplement: Supplementary file 5 — Additional file 5: Fig. S3. Evaluation of LPS contamination or inflammatory induction by individual AuNPs. [file 12951_2021_1203_MOESM5_ESM.tif]

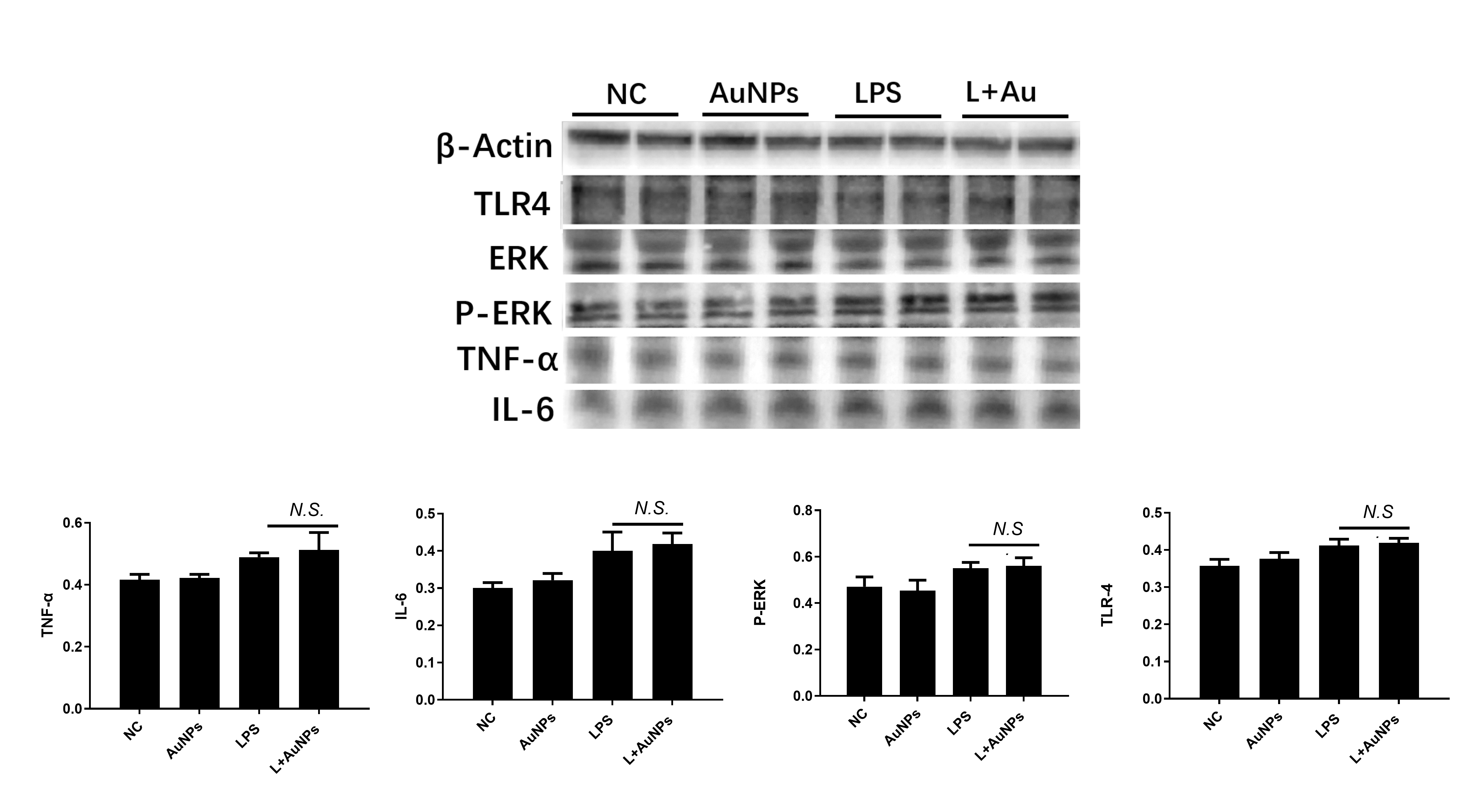

Supplement: Supplementary file 6 — Additional file 6: Fig. S4. The expression of inflammatory factors in the liver of mice was treated with AuNPs and LPS alone or together. [file 12951_2021_1203_MOESM6_ESM.tif]

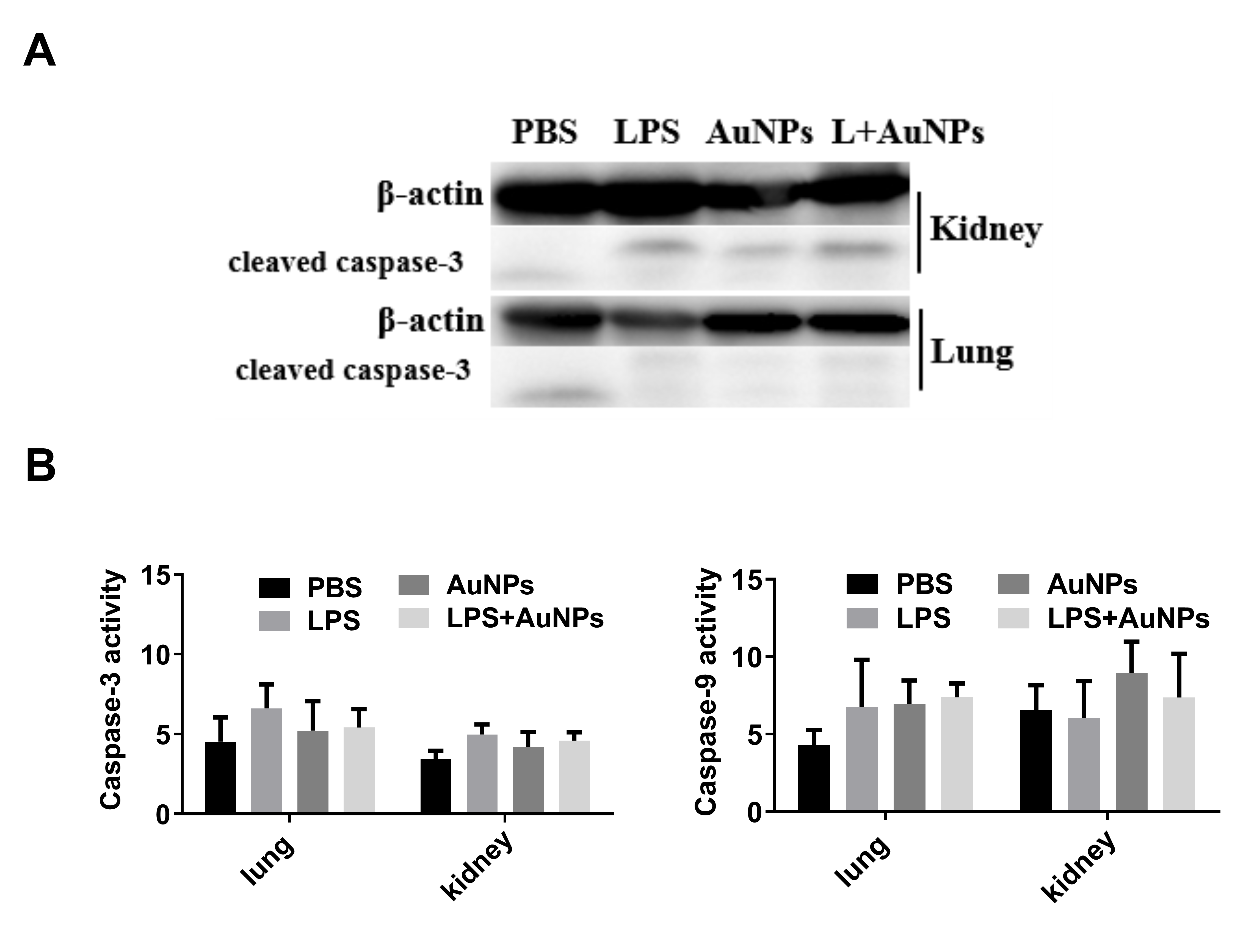

Supplement: Supplementary file 7 — Additional file 7: Fig. S5. Apoptosis in kidney and lung tissues by co-treatment of AuNPs and LPS. [file 12951_2021_1203_MOESM7_ESM.tif]

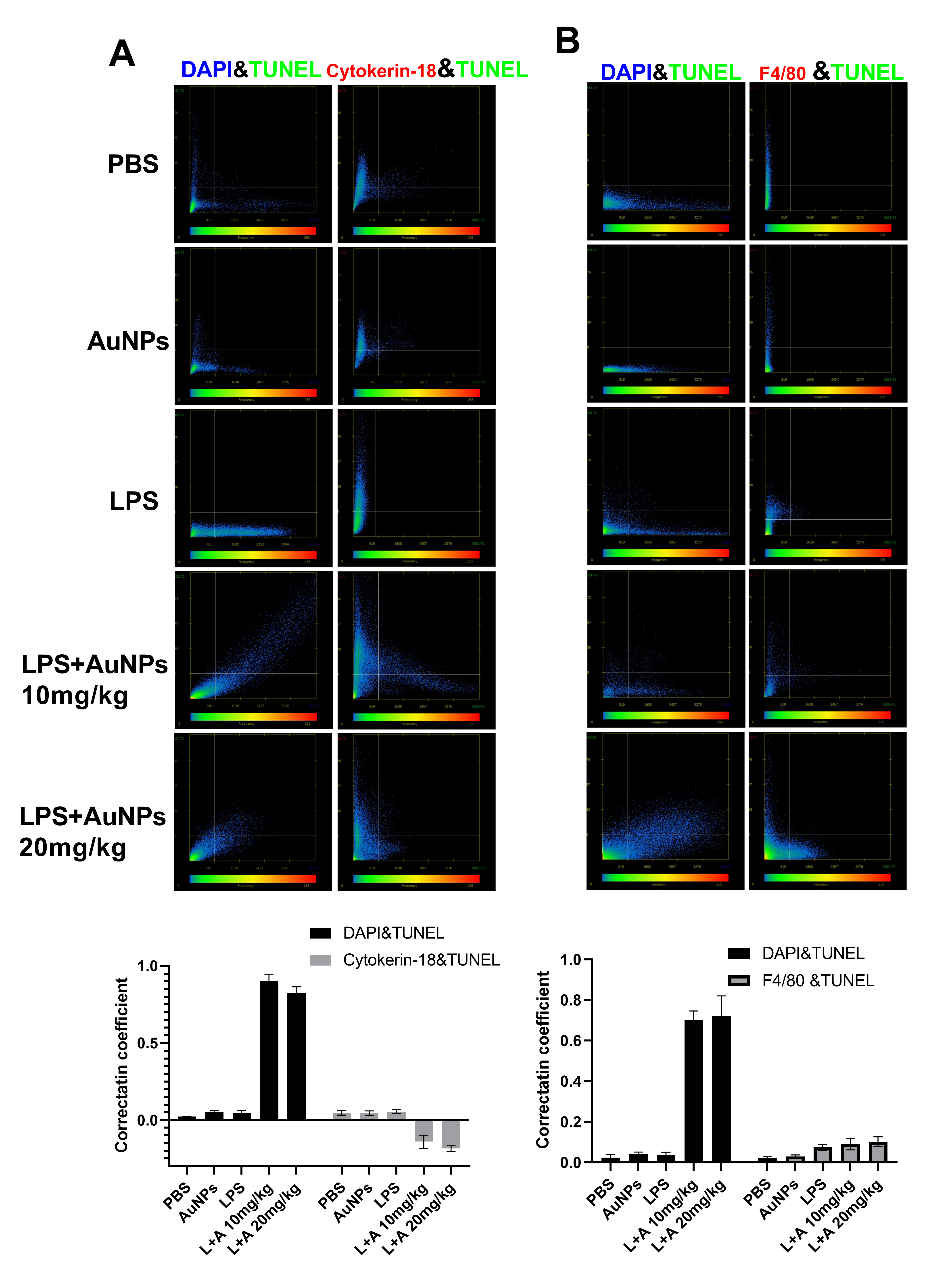

Supplement: Supplementary file 8 — Additional file 8: Fig. S6. Co-localization analysis of immunofluorescence images. [file 12951_2021_1203_MOESM8_ESM.tif]

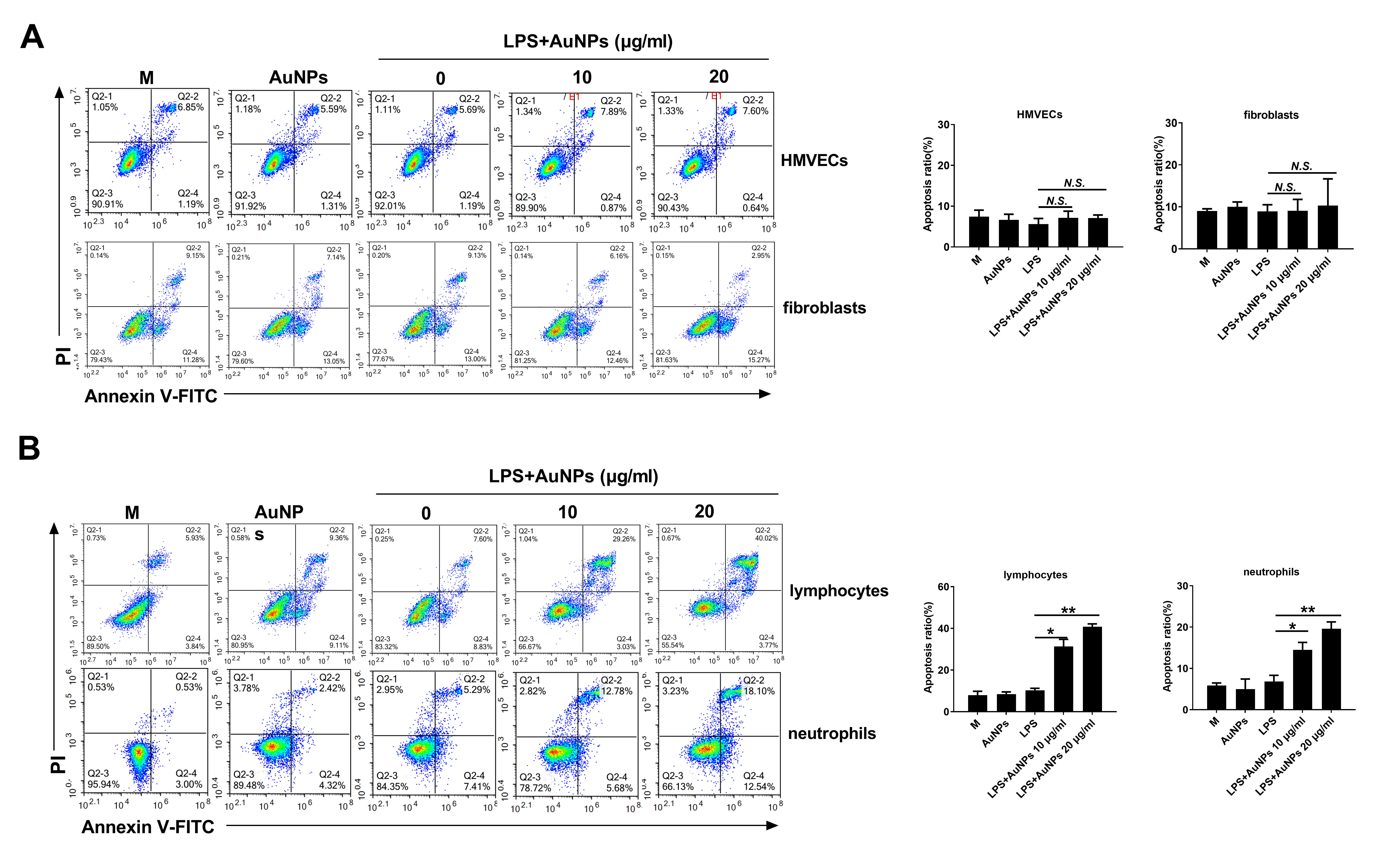

Supplement: Supplementary file 9 — Additional file 9: Fig. S7. Apoptosis in other parenchymal or non-parenchymal cells induced by AuNPs and LPS co-treatment. [file 12951_2021_1203_MOESM9_ESM.tif]

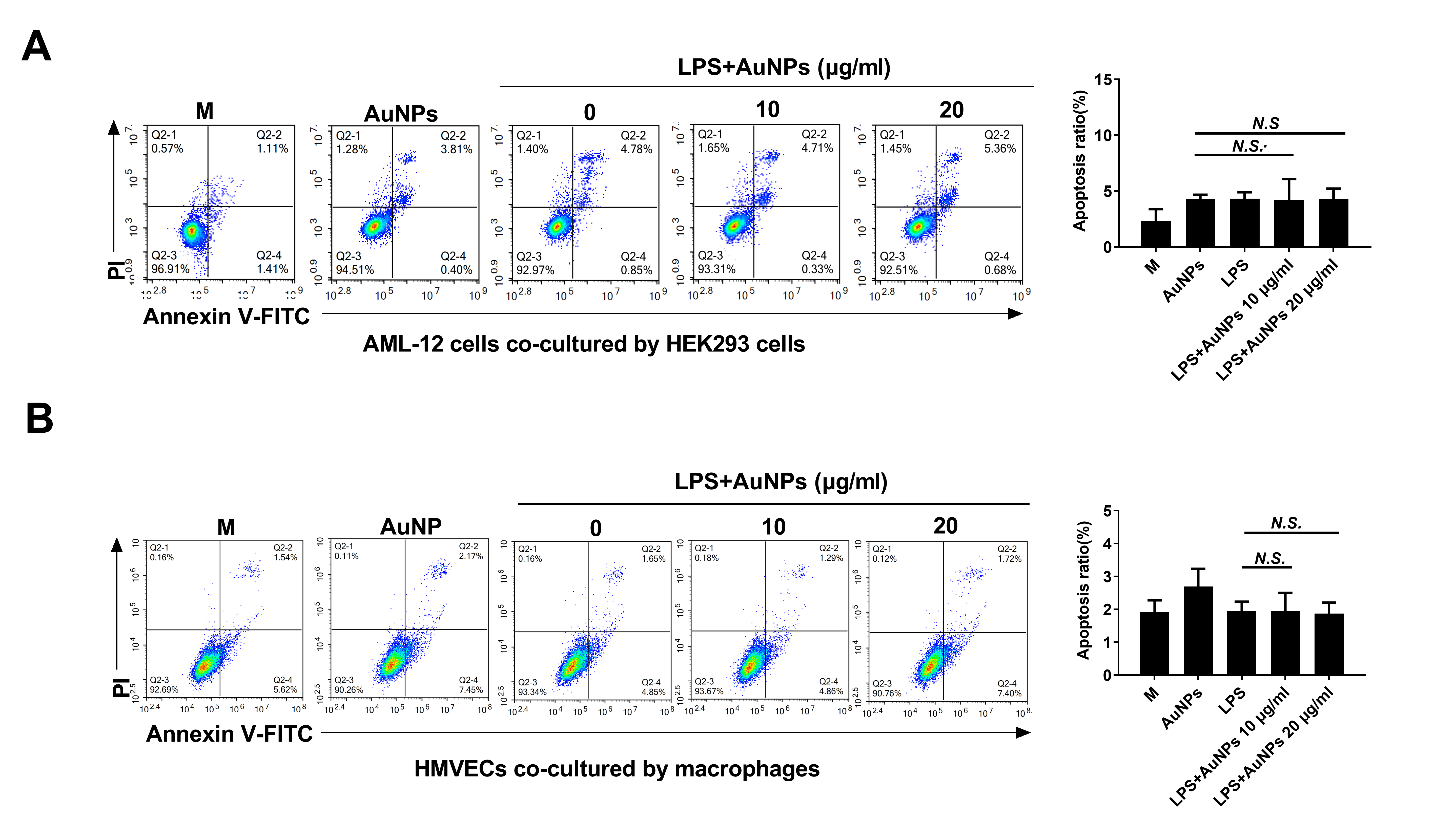

Supplement: Supplementary file 10 — Additional file 10: Fig. S8. Apoptosis in co-cultured cells treated by AuNPs and LPS. [file 12951_2021_1203_MOESM10_ESM.tif]

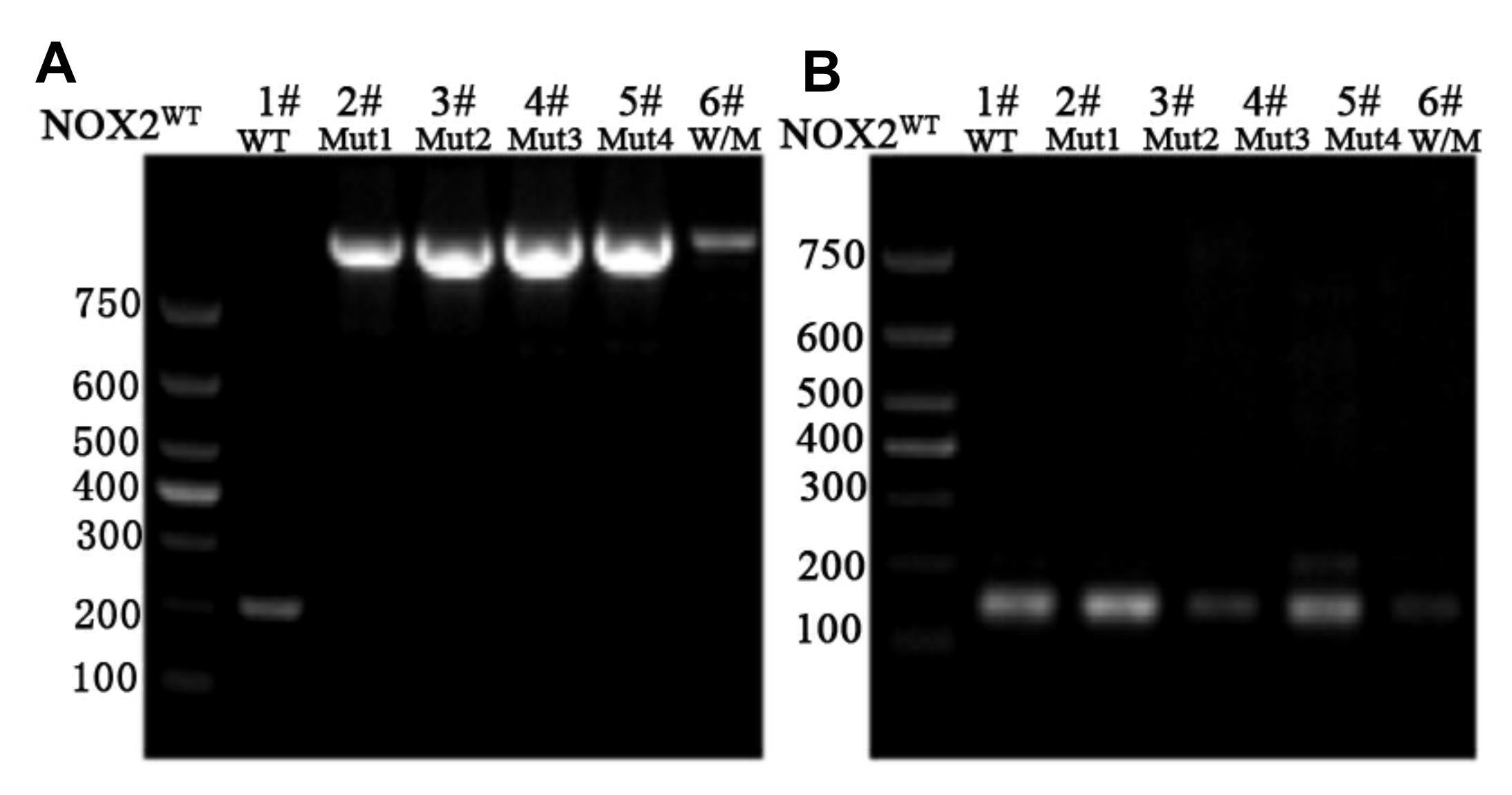

Supplement: Supplementary file 11 — Additional file 11: Fig. S9. Identification of NOX2 deficient mice. [file 12951_2021_1203_MOESM11_ESM.tif]

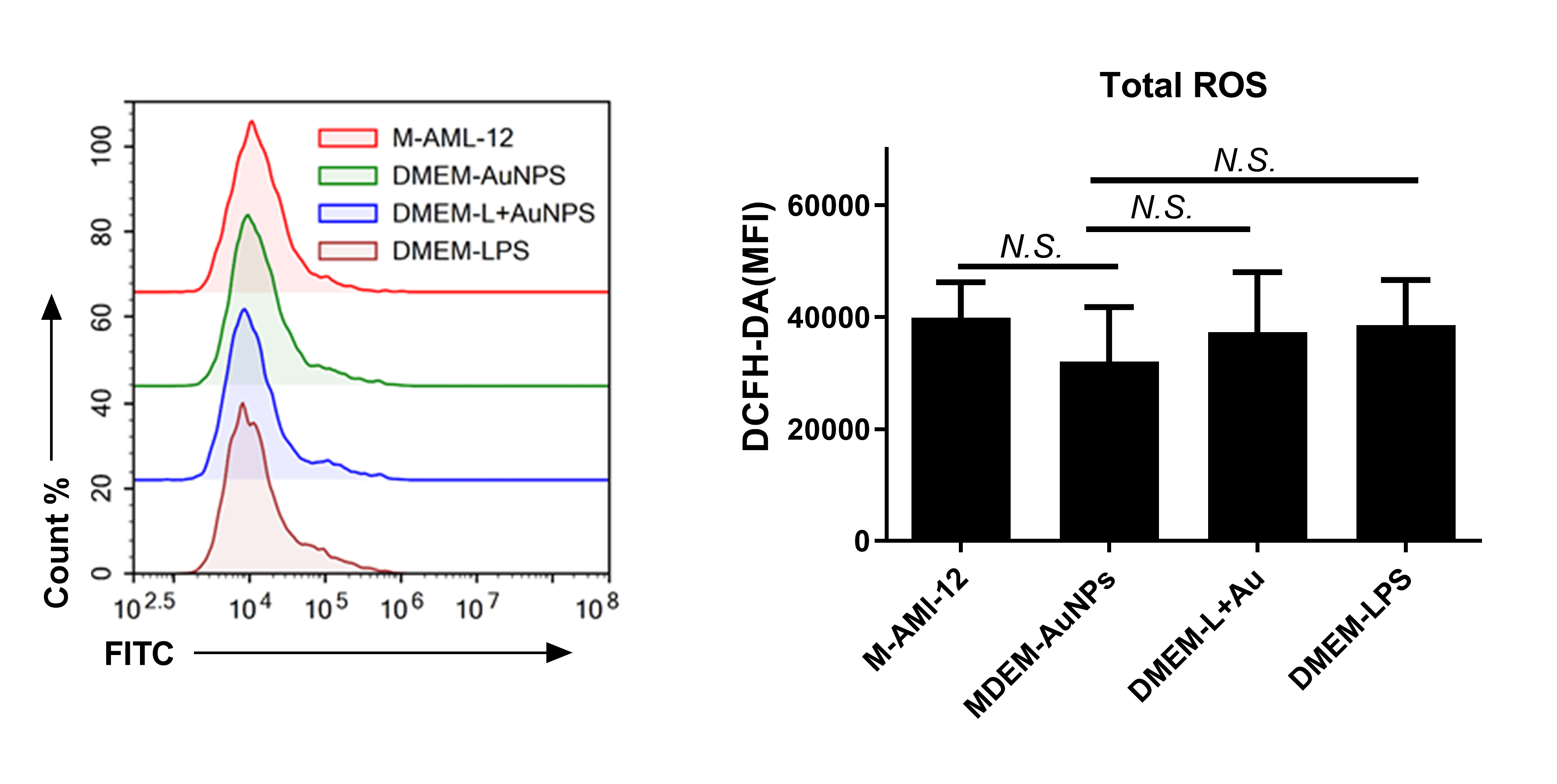

Supplement: Supplementary file 12 — Additional file 12: Fig. S10. ROS determination of AuNPs, LPS and LPS + AuNPs in DMEM. [file 12951_2021_1203_MOESM12_ESM.tif]

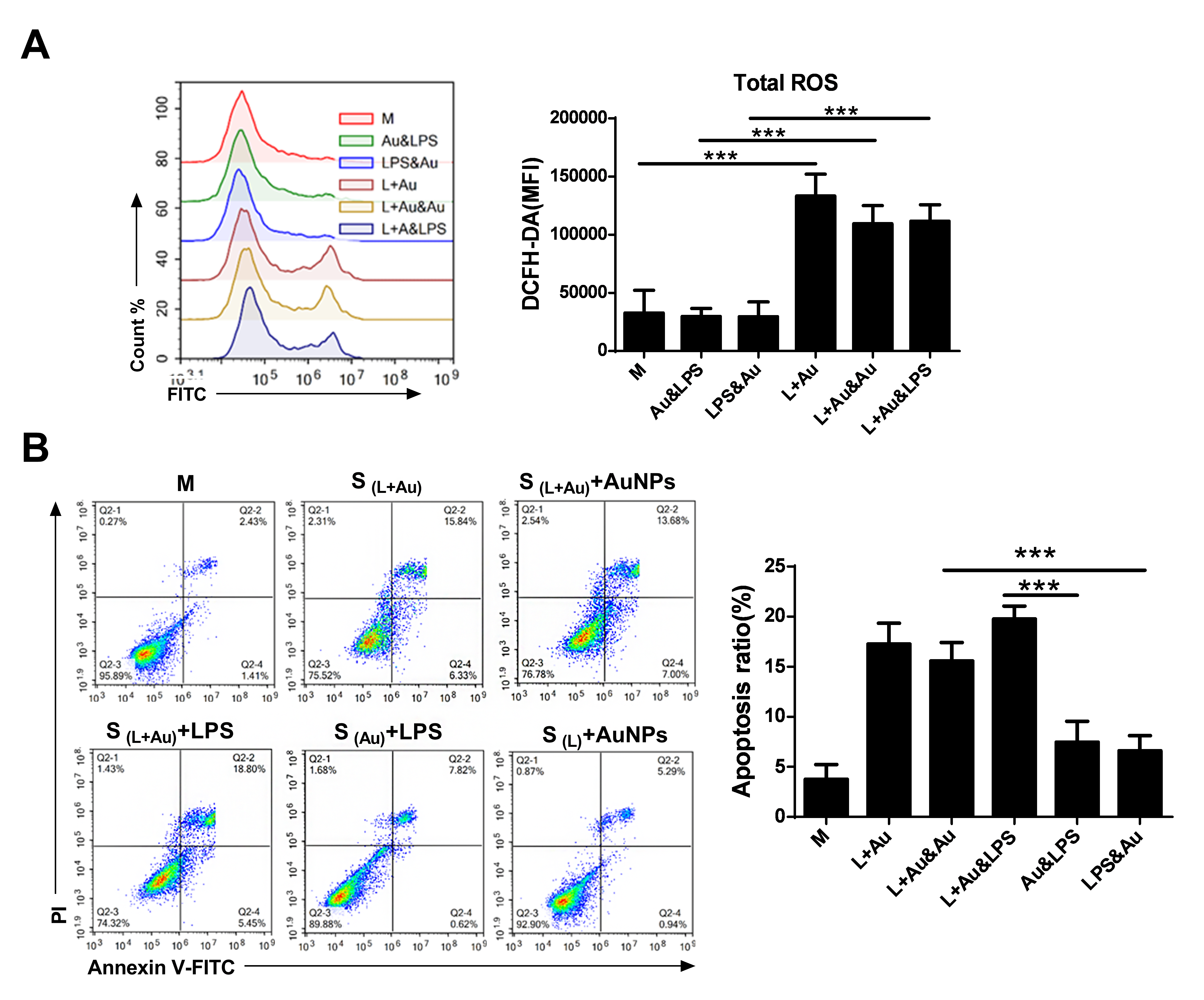

Supplement: Supplementary file 13 — Additional file 13: Fig. S11. Effects of LPS and AuNPS alone or co-cultured supernatant on ROS and apoptosis of AML-12 cells. [file 12951_2021_1203_MOESM13_ESM.tif]

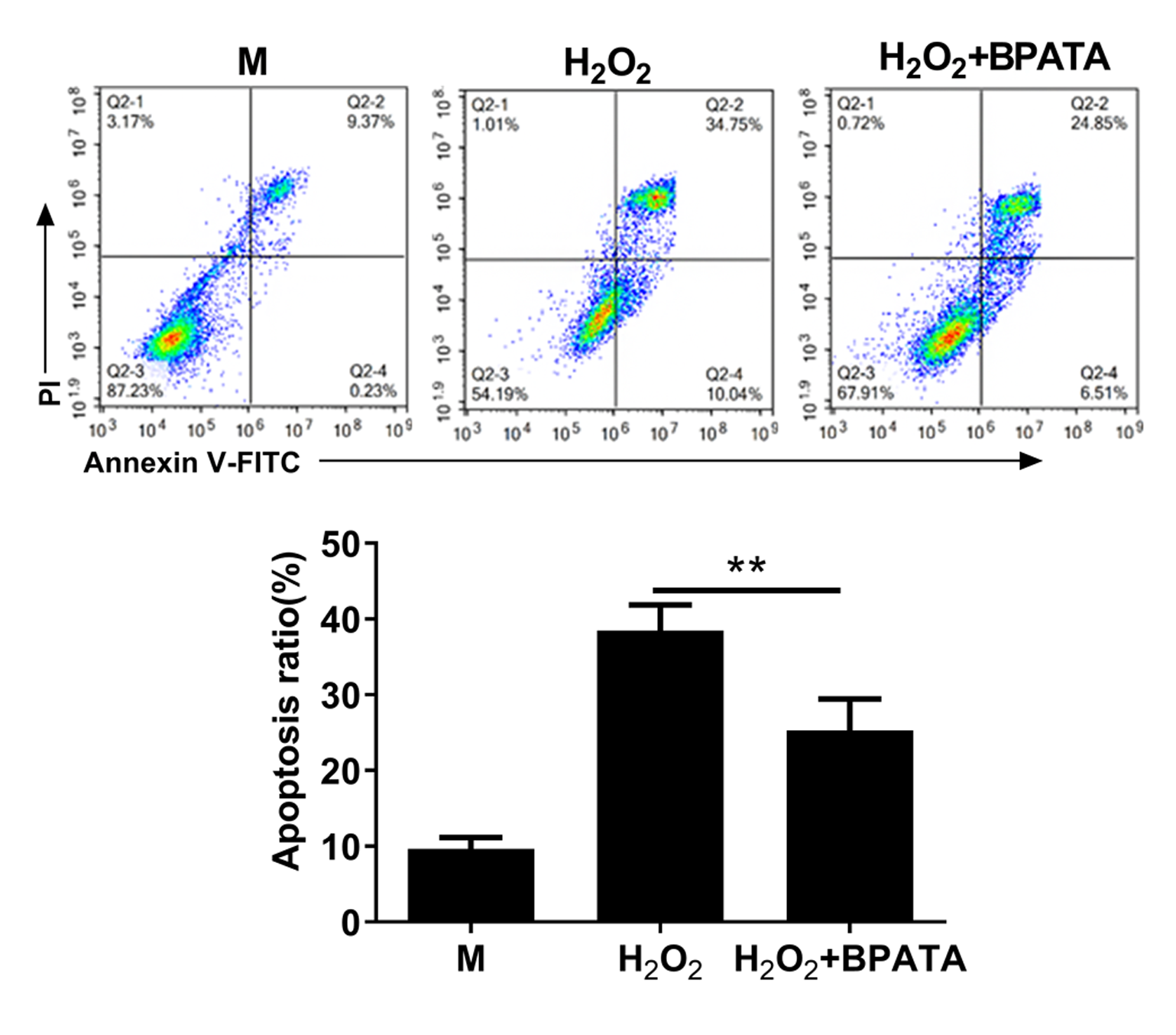

Supplement: Supplementary file 14 — Additional file 14: Fig. S12. The effect of BPATA on cell apoptosis caused by hydrogen peroxide. [file 12951_2021_1203_MOESM14_ESM.tif]
