# Supplementary material for: Acute exposure to gold nanoparticles aggravates lipopolysaccharide-induced liver injury by amplifying apoptosis via ROS-mediated macrophage-hepatocyte crosstalk
Source: J Nanobiotechnology. 2022 Jan 20;20:37. doi: 10.1186/s12951-021-01203-w (PMC8772144; doi:10.1186/s12951-021-01203-w)
Supplement: Supplementary file 2 — Additional file 2: Table S2. Physicochemical properties of AuNPs (10 μg/ml). [file 12951_2021_1203_MOESM2_ESM.docx]

**Table S2** Physicochemical properties of AuNPs (10 μg/ml) (mean ± SD, n = 3).

| AuNPs | SPR peak  (nm) | Hydrodynamic size (nm) | | | ζ Potential (mV) | | |
| --- | --- | --- | --- | --- | --- | --- | --- |
|  |  | H_2_O | PBS | DMEM | H_2_O | PBS | DMEM |
| 10 μg/ml | 514 | 10.2±0.4 | 21.4±2.7 | 25.2±2.4 | -14.2±2.1 | -15.2±2.8 | -9.2±1.9 |
