# Supplementary material for: Acute exposure to gold nanoparticles aggravates lipopolysaccharide-induced liver injury by amplifying apoptosis via ROS-mediated macrophage-hepatocyte crosstalk
Source: J Nanobiotechnology. 2022 Jan 20;20:37. doi: 10.1186/s12951-021-01203-w (PMC8772144; doi:10.1186/s12951-021-01203-w)
Supplement: Supplementary file 4 — Additional file 4: Table S3. Physicochemical properties of AuNPs (1 and 50 μg/ml). [file 12951_2021_1203_MOESM4_ESM.docx]

**Table. S3** Physicochemical properties of AuNPs (1 and 50 μg/ml) (mean ± SD, n =3).

| AuNPs | SPR peak  (nm) | Hydrodynamic size (nm) | | | ζ Potential (mV) | | |
| --- | --- | --- | --- | --- | --- | --- | --- |
|  |  | H_2_O | PBS | DMEM | H_2_O | PBS | DMEM |
| 1 μg/ml | 515 | 11.4±0.1 | 18.2±2.2 | 24.4±1.8 | -15.2±1.5 | -14.1±0.9 | -11.2±2.1 |
| 50 μg/ml | 514 | 11.5±0.2 | 19.5±3.2 | 26.5±2.2 | -16.5±2.2 | -14.5±1.8 | -10.2±2.0 |
