# Supplementary material for: Acute exposure to gold nanoparticles aggravates lipopolysaccharide-induced liver injury by amplifying apoptosis via ROS-mediated macrophage-hepatocyte crosstalk
Source: J Nanobiotechnology. 2022 Jan 20;20:37. doi: 10.1186/s12951-021-01203-w (PMC8772144; doi:10.1186/s12951-021-01203-w)
Supplement: Supplementary file 15 — Additional file 15: Table S1. Real-time PCR primers. [file 12951_2021_1203_MOESM15_ESM.docx]

**Table S1** Real-time PCR primers

| primer | Sense (5’-3’) | antisense (3’ - 5’) |
| --- | --- | --- |
| β-actin | TGTTACCAACTGGGACGACA | CTGGGTCATCTTTTCACGGT |
| SRA1 | ATTGGGAAATGAAGAACTGC | GGACTGACGAAATCAAGGAA |
